# Enhancing Gut Microbiome and Metabolic Health in Mice Through Administration of Presumptive Probiotic Strain *Lactiplantibacillus pentosus* PE11

**DOI:** 10.3390/nu17030442

**Published:** 2025-01-25

**Authors:** Ioanna Farmakioti, Electra Stylianopoulou, Nikistratos Siskos, Evangelia Karagianni, Dionysios Kandylas, Andreas Rafail Vasileiou, Fragkiski Fragkiskatou, Paraskevi Somalou, Alexandra Tsaroucha, Petros Ypsilantis, Panayiotis Panas, Yiannis Kourkoutas, George Skavdis, Maria E. Grigoriou

**Affiliations:** 1 Department of Molecular Biology & Genetics, Democritus University of Thrace, 68100 Alexandroupolis, Greece; ifarmaki@mbg.duth.gr (I.F.); ilstylian@mbg.duth.gr (E.S.); nsiskos@mbg.duth.gr (N.S.); evelinakaragianni@gmail.com (E.K.); sakis.kandilas@hotmail.com (D.K.); anvasile@mbg.duth.gr (A.R.V.); fragkiski.hfragkiskatou@yahoo.com (F.F.); psomalou@mbg.duth.gr (P.S.); ikourkou@mbg.duth.gr (Y.K.); gskavdis@mbg.duth.gr (G.S.); 2 Department of Medicine, Democritus University of Thrace, 68100 Alexandroupolis, Greece; atsarouc@med.duth.gr (A.T.); pipsil@med.duth.gr (P.Y.); 3 QLC, NEO Patron Athinon 57, 26442 Patras, Greece; panas@qlc.gr

**Keywords:** *Lactiplantibacillus pentosus* PE11, overall health, gut microbiota, triglycerides levels, alanine aminotransferase levels

## Abstract

**Background:** Over the past decade, probiotics have gained increasing recognition for their health benefits to the host. While most research has focused on the therapeutic effects of probiotics in the treatment of various diseases, recent years have seen a shift towards exploring their role in enhancing and supporting overall health. **Methods:** In this work, we have studied the effects of a novel potential probiotic strain, *Lactiplantibacillus pentosus* PE11, in healthy mice following a six-week dietary intervention. The assessment included monitoring the general health of the animals, biochemical analyses, profiling of the gut and fecal microbial communities, and gene expression analysis. **Results:** Our results showed that the administration of *Lactiplantibacillus pentosus* PE11 led to changes in the composition of the fecal microbiome, specifically an increase in the Firmicutes/Bacteroidetes ratio and in the relative abundance of the Lachnospiraceae, Ruminococcaceae, and Rikenellaceae families. Reduced *Tnf* expression and elevated *Zo1* expression were also observed in the cecum, pointing to anti-inflammatory properties and improved intestinal barrier integrity. Additionally, a significant reduction in triglycerides and alanine aminotransferase levels—within physiological ranges—was observed, along with a trend toward decreased total cholesterol levels. **Conclusions:** These findings suggest that in healthy mice, *Lactiplantibacillus pentosus* PE11 has the potential to positively influence gut microbiome structure and metabolism, thereby supporting improved overall health.

## 1. Introduction

The intestinal microbiota is a complex and diverse community of microorganisms residing in the gastrointestinal (GI) tract comprising hundreds of species of bacteria, fungi, archaea, protozoa, and viruses [1,2,3]. Among these, bacterial species are the most predominant [4]. The structure of the gut microbial community varies across different segments of the GI, reflecting the distinct environmental conditions in each region. Microbial abundance and diversity gradually increase along the GI, from the relatively low levels in the stomach to the dense and diverse populations found in the colon [1,2,5]. Gut microbiota maintains a symbiotic relationship with the host and, along with its metabolites, has a crucial role; its primary functions include nutrient metabolism, xenobiotic and drug metabolism, vitamin synthesis, and protection against pathogens [1,6]. Additionally, the gut microbiota contributes to the development of the immune system and the development and modulation of the nervous system, as well as the maintenance of the integrity of the epithelial barrier [1,6]. Due to its extensive connections with various organs via neural, endocrine, humoral, immunological, and metabolic pathways, the gut microbiota was recently recognized as a “vital organ” [7]. Numerous human diseases, including gastrointestinal, metabolic, cardiovascular, neurodevelopmental, and neurodegenerative disorders were linked to specific changes in the structure of the gut microbiota—a state known as dysbiosis [3,7,8,9].

According to the Food and Agriculture Organization of the United Nations (FAO) and the World Health Organization (WHO), probiotics are “live microorganisms which when administered in adequate amounts confer a health benefit on the host” [10]. Over the past 20 years, probiotics were the focus of intensive research. Several bacterial strains, of the *Pediococcus*, *Lactococcus*, *Enterococcus*, *Streptococcus*, *Propionibacterium*, *Bacillus, Lactobacillus*, and *Bifidobacterium* genera, commonly found in fermented foods, were characterized for their probiotic properties [11,12]. Yeast strains of the genus *Saccharomyces* have also been characterized as probiotics [11]. A large body of evidence points to the beneficial effects of probiotic administration in managing various diseases, for instance intestinal inflammatory diseases, such as Crohn’s Disease and inflammatory bowel disease (IBD) or metabolic diseases, such as diabetes and obesity [3,11]; in these conditions, the positive effects of probiotic administration include strengthening the gut barrier, suppressing pathogen growth, and modulating the immune system [4,12]. Moreover, probiotics contribute significantly to the modulation of lipid and bile salt metabolism, the synthesis of vitamin K, riboflavin and folate, enzyme activity, toxin neutralization, and intestinal electrolyte absorption [4,12]. Most of the studies on probiotics focus on their role in disease management; relatively little is known, however, about strains that positively affect healthy individuals and the potential role probiotics may play in preserving overall health. A recent review evaluated the evidence supporting the efficacy of probiotics in preventing various diseases and promoting overall health in generally healthy populations, and while the findings highlighted promising potential, scientists concluded that the research remains insufficient to recommend probiotics for routine use in healthy individuals due to the emerging nature of the field as well as unresolved questions about strains and dosages [13]. Probiotic properties are strain-specific, as different strains have different mechanisms of action [14]. A strain that is effective in managing a specific disorder may not have positive effects on healthy individuals. Unlike the study of probiotics in disease management, where outcomes are evaluated through symptom improvement, exploring the role of probiotics in healthy individuals presents unique challenges. Notably, the definition of a “normal” or “healthy” human gut microbiota remains a subject of debate [15]. Given the established diversity in the microbial communities of healthy populations—shaped by factors such as genotype, gender, diet, and age [4]—it is unrealistic to expect uniform effects from different probiotic strains or to assume that a particular strain will have identical effects across individuals. Thus, clinical studies in healthy individuals are needed to demonstrate the ability of specific probiotic strains to reduce the risk of adverse health outcomes and clarify their beneficial effects to support their recommendation as a preventive therapy. In this vein, studies in healthy animals are an essential prerequisite, as they can help identify strains that offer health benefits, evaluate their impact on overall well-being, and provide critical data before human trials.

*Lactiplantibacillus pentosus* PE11 (*Lb. pentosus* PE11) was isolated from olive fruit, classified and characterized through Whole Genome Sequencing analysis [16]. Notably this species was included in the European Food Safety Authority’s (EFSA) QPS (Qualified Presumption of Safety) list. In vitro tests assessing the probiotic functional properties revealed that *Lb. pentosus* PE11 demonstrated robust probiotic characteristics, meeting the established standards for probiotics [16]. In this study, we investigate the effects of *Lb. pentosus* PE11 on healthy mice following a dietary intervention with a focus on GI microbiota, as well as biochemical and molecular markers of overall health. *Lb. pentosus* PE11 demonstrated probiotic potential by increasing butyrate-producing families like Lachnospiraceae and reducing triglycerides and alanine aminotransferase levels, suggesting benefits for metabolic and liver health. Region-specific effects on the expression of genes involved in intestinal barrier integrity and anti-inflammatory activity in the cecum suggest its role in promoting gut, and, when considered together with previous data, overall health.

## 2. Materials and Methods

### 2.1. Preparation of Bacterial Cultures

*Lb. pentosus* PE11 [16] was grown anaerobically on a synthetic medium (20 g/L glucose, 25 g/L yeast extract, 2 g/L KH_2_PO_4_, 6 g/L CH_3_COONa, 0.3 g/L MgSO_4_, and 0.05 g/L MnSO_4_) at 37 °C for 24 h. Freeze-dried cells were prepared as previously described [16]. Cell viability during storage was confirmed by microbiological analysis.

### 2.2. Animals

Three-month-old male C57BL/6 mice were housed in individually ventilated cages with free access to sterile laboratory chow (Mucedola, Italy, type 4RF25) and water. The mice were maintained at the Laboratory of Experimental Surgery and Surgical Research, Department of Medicine, Democritus University of Thrace (EL71 BIO exp 1) under a 12 h light/dark cycle at 21 ± 2 °C with relative humidity at 55 ± 10%. G-power analysis was conducted to estimate the number of mice required for each experimental group. Mice were randomly divided into two groups (n = 5 per group), in the same cage per treatment. Following a one-week adaptation, one group received oral gavage with a suspension of 10⁹ CFU of *Lb. pentosus* PE11 in 200 μL sterile phosphate-buffered saline (PBS, Invitrogen, Waltham, MA, USA) (LB group), while the second group received 200 μL of sterile PBS (PBS group, control). To minimize stress during gavage, trained personnel handled the mice gently, performed the procedure consistently at the same time daily, and used appropriately sized tubes. Administration volumes were within species-appropriate limits, and animals were monitored throughout to ensure their well-being. The *Lb. pentosus* PE11 suspension was freshly prepared for each dose by resuspending freeze-dried bacterial cells in 200 μL PBS to achieve a final concentration of 5 × 10^9^ CFU/mL. Treatments were administered daily for the first week and every other day for the subsequent five weeks. Body weight was recorded weekly by trained personnel who were blinded to the group assignments of the mice. Fecal samples were collected at the start of the experiment (Day 0) as well as at the end of the six-week experimental period (the day after the last gavage) and were stored at −80 °C until DNA isolation.

Mice were sacrificed via cervical dislocation, and blood was collected by cardiac puncture. All subsequent steps were performed on ice. Large and small intestines were dissected out and segmented according to the regions of interest. Luminal contents from each region were immediately collected by gently pressing the corresponding segment with forceps. Samples were stored at −80 °C for subsequent analyses. After removing residual intestinal contents by rinsing the tissues with cold PBS, each biopsy was divided into two equal segments. One segment consistently from the same region for each mouse was immediately stored at −80 °C for subsequent gene expression analysis, while the second was immersed in 4% *w*/*v* paraformaldehyde (PFA) in PBS for 24 h at 4 °C for subsequent histological analysis.

All animal experiments were conducted in accordance with Directive 2010/63 of the European Parliament and Council of 22 September 2010, which was the legislation in force at the time. The protocol was approved by the Animal Care and Use Committee of the Prefecture of Evros, Thrace, Greece, under permit number 36662/118 (08/02/2022).

### 2.3. Serum Biochemical Analyses

Blood samples were centrifuged at 3000 rpm for 15 min at 4 °C. Serum was collected and stored at −80 °C until further use. Serum levels of alanine aminotransferase (ALT), alkaline phosphatase (ALP), blood urea nitrogen (BUN), creatinine (CREA), high-density lipoprotein (HDL), low-density lipoprotein (LDL), total cholesterol (TC), and triglycerides (TGs) were analyzed at an accredited laboratory (LABnet, Thessaloniki, Greece).

### 2.4. Histology

Fixed tissues were washed with PBS, cryoprotected in 30% *w*/*v* sucrose in PBS at 4 °C, embedded in Tissue Freezing Medium (Leica, Wetzlar, Germany), and sectioned at 12 µm using a Leica CM1900 UV cryostat (Leica, Wetzlar, Germany). Tissue sections were post fixed in 4% *w*/*v* PFA in PBS for 10 min, washed in water for 2 min, and stained with hematoxylin (Applichem, Darmstadt, Germany) for 1 min. Following a rinse in water for 5 min to remove excess stain, the sections were differentiated in 1% *v*/*v* hydrogen chloride (Applichem, Darmstadt, Germany) in 70% *v*/*v* ethanol for 5 s and then washed in water for approximately 5 min, followed by a wash step in 95% *v*/*v* ethanol for 1 min. Sections were subsequently stained with eosin (Applichem, Darmstadt, Germany) for 1 min, washed in water for 20–30 s, dehydrated through alcohol series (70% *v*/*v* ethanol for 5 s, 95% *v*/*v* ethanol for 5 s twice and absolute ethanol for 1 min), cleared in xylene (Applichem, Darmstadt, Germany), and mounted in DPX Mountant for histology (Sigma-Aldrich, St. Louis, MO, USA). Samples were analyzed with a Leica DM5500 B microscope (Leica Microsystems, Wetzlar, Germany). Images were obtained using camera software (LAS v4.13, Leica Microsystems) and image panels were designed using the GIMP version 2.10.38 (https://www.gimp.org, accessed on 15 November 2024). Villus height and crypt depth were measured in ImageJ version 2.9.0/1.53t (https://imagej.nih.gov, accessed on 20 October 2024). All the sections were blindly evaluated by two different observers.

### 2.5. Gene Expression Analysis

RNA isolation and cDNA synthesis were carried out using standardized protocols, with procedures conducted simultaneously across samples of both groups to eliminate potential bias. Total RNA was isolated from biopsies using Trizol Reagent (TR, Thermo Fisher Scientific, Waltham, MA, USA), following the manufacturer’s instructions. Tissues were lysed and homogenized using 1 mL TR per 50–100 mg of tissue. Then, 0.2 mL chloroform (Sigma-Aldrich, St. Louis, MO, USA) per mL of TR was added to the lysates, and the samples were centrifuged at 12,000 g for 15 min at 4 °C. The aqueous phase containing RNA was carefully separated and RNA was precipitated with 0.5 mL isopropanol (Sigma-Aldrich, St. Louis, MO, USA) per mL of TR, followed by washing the pellet with 1 mL 75% *v*/*v* ethanol (Scharlab S.L., Barcelona, Spain) per mL of TR. Finally, the RNA pellet was briefly air-dried and dissolved in RNase-free water (Thermo Fisher Scientific, Waltham, MA, USA). The concentration and purity of the RNA were assessed using a NanoDrop Spectrophotometer (Thermo Fisher Scientific, Waltham, MA, USA) and its integrity was assessed by loading 100 ng of RNA on a 1.5% *w*/*v* agarose gel (UltraPure Agarose, Invitrogen, Carlsbad, CA, USA). RNA was stored at −80 °C.

cDNA synthesis was performed using the Maxima H Minus First Strand cDNA Synthesis Kit with dsDNase (Thermo Fisher Scientific, Waltham, MA, USA), according to the manufacturer’s instructions. A total of 5 μg of RNA were incubated with dsDNase initially at 37 °C for 2 min and then at 55 °C for 5 min. Then, 100 pmol oligo (dT)18 primer, 100 pmol random hexamer primer, and 0.5 mM of dNTP Mix were added to the reaction and the sample was incubated at 65 °C for 5 min. Subsequently, 4 µL of 5X RT Buffer and 1 µL Maxima H Minus Enzyme Mix were added to the reaction and cDNA synthesis was performed by incubation first at 25 °C for 10 min and then at 50 °C for 30 min. The reaction was stopped by incubating the sample at 85 °C for 5 min. The cDNA was stored at −20 °C.

Primers that amplify part of the coding sequences of the following genes were designed using the NCBI Primer Blast tool, based on the cDNA sequences published in GenBank: *Zo1* (Zona Occludens 1), *Ocln* (Occludin), *Jama* (Junctional Adhesion Molecule A), *Tlr2* (Toll-Like Receptor 2), *Muc2* (Mucin 2), *Tgfb* (Transforming Growth Factor Beta) *Tnf* (Tumor Necrosis Factor), *Il1b* (Interleukin 1 Beta), *Il6* (Interleukin 6), *Il10* (Interleukin 10), *Fiaf* (Fasting-Induced Adipose Factor), *Fitm2* (Fat Storage Inducing Transmembrane Protein 2), *Sert* (5-hydroxytryptamine -Serotonin- Transporter), and *Canx* (Calnexin). The sequence, product length, and melting temperature (Tm) for each primer pair are listed in Table S1.

For Quantitative Polymerase Chain Reaction (qPCR), a 1:25 or 1:125 dilution of the cDNA was used. The same dilution was used across all cDNAs for each target gene. qPCR was conducted using a StepOne™ (Thermo Fisher Scientific, Waltham, MA, USA) thermocycler under the following conditions: initial denaturation at 95 °C for 2.5 min, followed by 40 cycles of denaturation at 95 °C for 10 s, annealing at the appropriate Tm for 20 s, and elongation at 72 °C for 10 s. The qPCR reaction volume was 20 μL, consisting of 10 μL KAPA SYBR FAST qPCR Master Mix (2×) (Sigma-Aldrich, St. Louis, MO, USA), 0.5 μM forward primer, 0.5 μM reverse primer, and 5 μL diluted cDNA.

At the end of the reactions, the melting curves were analyzed to confirm amplification specificity. Baseline correction and PCR efficiency were determined for each reaction using the LinRegPCR program (v.2021.2) [17]. Efficiency-weighted Ct values were calculated for each pair of data points (Ct and PCR efficiency), along with the mean efficiency-weighted Ct value from the three technical replicates. Expression levels for each target gene were normalized to *Canx*, which was used as the reference gene. Fold changes in the expression of the target genes were calculated using the samples of the PBS group as reference [18]. Parametric statistics were conducted on ΔΔCt values, as this approach is considered more robust and reliable compared to performing statistical analyses directly on fold changes, which is generally deemed invalid [18]. A *p*-value less than 0.05 demonstrates that ΔΔCt was statistically different from 0 and thus the fold change was significantly different from 1.

### 2.6. Analysis of Microbial Communities

#### 2.6.1. DNA Extraction

Metagenomic DNA isolation was performed using the NucleoSpinStool Mini Kit (Macherey-Nagel, Düren, Germany) according to the manufacturer’s instructions. Samples were homogenized with Precellys Evolution Homogenizer (Bertin Instruments, Montigny-le-Bretonneux, France) (3 cycles at 6300 rpm for 30 s with 30 s interval between cycles). A NanoDrop Spectrophotometer was used to assess purity and concentration of the isolated DNA; integrity was assessed by gel electrophoresis on a 1% *w*/*v* agarose gel. The samples for both groups were processed simultaneously to eliminate potential bias.

#### 2.6.2. Amplicon-Based Next-Generation Sequencing

The following procedures were performed using standardized protocols, with procedures carried out simultaneously across samples of both groups to mitigate potential bias. Amplicon-Based Next-Generation Sequencing (NGS) was employed to analyze the structure of microbial communities. The V4 hypervariable region of the *16SrRNA* locus, approximately 290 bp in length, was amplified using the primers 5′-GTGCCAGCMGCCGCGGTAA-3′ (forward) and 5′GGACTACHVGGGTWTCTAAT-3′ (reverse). For the Polymerase Chain Reaction (PCR), 50 ng of metagenomic DNA were used in a reaction of 20 μL with 10 μL of KAPA SYBR FAST qPCR Master Mix and 0.2 μΜ of each primer. The cycling conditions were as follows: initial denaturation at 95 °C for 5 min, followed by 25 cycles of denaturation at 95 °C for 30 s, annealing at 58 °C for 40 s, and elongation at 72 °C for 40 s, with a final elongation step at 72 °C for 5 min.

The PCR products were analyzed on a 2% *w*/*v* agarose gel, cleaned-up with NucleoMag NGS Clean-up and Size Select magnetic beads (Macherey-Nagel, Düren, Germany), at a volume ratio of DNA to beads of 1/1.8 and quantified using Qubit 4 Fluorometer (Thermo Fisher Scientific, Waltham, MA, USA) with the Qubit dsDNA HS Assay Kit (Thermo Fisher Scientific, Waltham, MA, USA). Library preparation and sequencing were performed on the ION Torrent S5 platform as previously described [19].

#### 2.6.3. Data Analysis

Torrent Suite software (Thermo Fisher Scientific, Waltham, MA, USA) was used to generate UBAM files of the raw data, excluding polyclonal, low-quality, and low-signal reads. The UBAM files were converted to FASTA format using Samtools (v.1.13) [20].

The initial steps of the analysis were performed with Mothur (v.1.45.3) [21], following previously described protocols [19]. Τhe same steps were applied across all FASTA files. An initial quality control was performed to filter out reads with ambiguous bases, homopolymers, and improper lengths. The alignment of the reads and the classification of them into Operational Taxonomic Units (OTUs) were conducted using the bacterial Greengenes (v.13_8_99) reference database. Classified reads with at least 97% sequence similarity were clustered into the same OTU. Low-abundance OTUs were filtered out using a 0.1% threshold with a 25% prevalence rule, implemented via the online platform MicrobiomeAnalyst (https://www.microbiomeanalyst.ca/, accessed on 6 September 2024) [22]. Alpha- and beta-diversity analyses were conducted, including the calculation of Shannon and Simpson diversity indices, rarefaction and Shannon curves, Principal Coordinates Analysis (PCoA) based on Bray–Curtis distance, and Permutational Multivariate Analysis of Variance (PERMANOVA). These analyses were carried out using the PAST (PAleontological STatistics) software package (v.4.03) [23]. Graphs displaying the median relative abundance of each group at the phylum and family levels were generated using GraphPad Prism 9.0 (GraphPad Software, San Diego, CA, USA).

### 2.7. Statistical Analysis

Differences in animal weight, biochemical parameter concentrations, colonic and cecal crypt depth, ileal villus height, gene expression levels, diversity indices, and relative abundances of bacterial taxa between groups were assessed using either an unpaired *t*-test (two-tailed) or a Mann–Whitney U-test (two-tailed). The *t*-test was applied to data with a normal distribution, while the Mann–Whitney U-test was used for non-normally distributed data. A *p*-value of less than 0.05 was considered statistically significant. Statistical analyses were performed using GraphPad Prism 9.0 (GraphPad Software, San Diego, CA, USA). Effect sizes were calculated using JASP software package (v.0.19.3). For the *t*-test, effect size was given by Cohen’s d (d), while for the Mann–Whitney U-test, it was given by the rank biserial correlation (r). A small effect was indicated by d values between 0.20 and 0.50, or r values between 0.10 and 0.30, an intermediate effect by d values between 0.50 and 0.80, or r values between 0.30 and 0.50 and a large effect by d values of 0.80 or higher, or r values between 0.50 and 1.00. The sign of effect sizes indicates the direction of the effect.

## 3. Results

### 3.1. Body Weight

Body weight did not differ significantly between the *Lb. pentosus* PE11-treated and control mice throughout dietary intervention (Figure S1A, Table S2). Similarly, no significant difference in total body weight gain was observed at the end of the intervention (Figure S1B, Table S2). These findings indicate that *Lb. pentosus* PE11 administration had no impact on growth performance.

### 3.2. Histological Analysis

Histological evaluation did not reveal any alterations (Figure S2). Moreover, no significant differences in the colonic and cecal crypt depth or in the height of the ileal villi were observed between the *Lb. pentosus* PE11-treated and control mice (Figure S3).

### 3.3. Analysis of Fecal Microbial Community

At a 97% similarity threshold, analysis of the V4 amplicon of *16S rRNA* from the samples identified 75 OTUs at the family level. After applying a 0.1% abundance filter combined with a 25% prevalence rule, 29 bacterial OTUs were retained (Tables S3 and S4), while 46 low-abundance OTUs were excluded. Rarefaction analysis confirmed that sequencing depth was sufficient to capture the microbial diversity within the samples (Figures S4 and S5).

**Figure 1:**
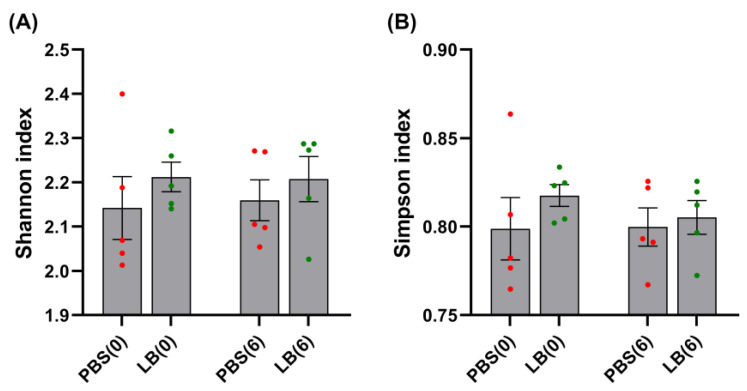
Shannon (**A**) and Simpson (**B**) diversity indices. Data are presented as mean ± SEM. PBS(0): control group—Day 0; PBS(6): control group—end of the intervention; LB(0): *Lb. pentosus* PE11 group—Day 0; LB(6): *Lb. pentosus* PE11 group—end of the intervention. *p* > 0.05, not indicated.

Alpha diversity did not vary across datasets with no significant differences observed in the Shannon and Simpson indices between the two groups either at the start or the end of the experiment (Figure 1).

**Figure 2:**
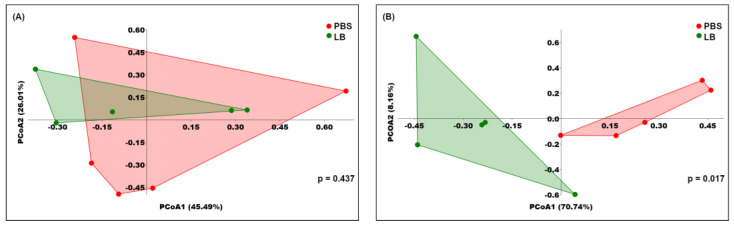
PCoA based on Bray–Curtis distance for fecal microbiota before (**A**) and after six-week intervention (**B**). Numbers in brackets indicate percentage of variance explained by corresponding coordinates (PCoA1 and PCoA2). *p*-Value was calculated using PERMANOVA. Each point on graph represents microbial community of fecal sample of individual mouse.

PCoA based on Bray–Curtis distance revealed no significant differences in the fecal microbiome structure between the two groups at the onset of the intervention (Day 0) (Figure 2A). However, after the six-week administration of *Lb. pentosus* PE11, a notable difference emerged (Figure 2B), indicating that the relative abundance of certain bacterial taxa was altered during the intervention.

**Figure 3:**
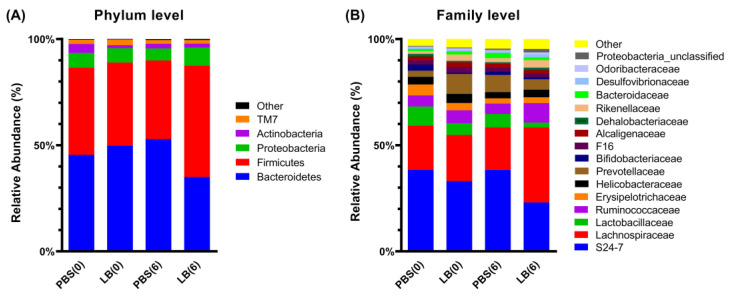
Relative abundance graphs of bacterial OTUs at phylum (**A**) and family (**B**) levels. “Other” represents bacterial taxa with median relative abundance of less than 1% in both groups. PBS(0): control group—Day 0; PBS(6): control group—end of the intervention; LB(0): *Lb. pentosus* PE11 group—Day 0; LB(6): *Lb. pentosus* PE11 group—end of intervention.

In all samples, the two dominant phyla were Bacteroidetes (22.87–54.43%) and Firmicutes (31.75–64.96%), collectively accounting for over 83% of the total bacterial population. Proteobacteria (3.82–9.03%) and Actinobacteria (0.90–5.12%) were also present. At Day 0, no significant differences were observed at either the phylum or family level between the PBS and LB groups (Figure 3A,B and Table S3). However, after intervention, at the phylum level, the relative abundance of Firmicutes was significantly higher in the LB group compared to the PBS group (51.15% vs. 36.66%) (*p* = 0.016, r = 0.920, 95% CI for r: 0.685, 0.982), while Bacteroidetes were significantly more abundant in the PBS group (52.38% vs. 34.16%) (*p* = 0.008, r = −1, 95% CI for r: −1, −1) (Figure 3A, Table S4). Consequently, the Firmicutes-to-Bacteroidetes (F/B) ratio was significantly higher in the LB group than in the PBS group (1.50 vs. 0.70) (*p* = 0.016, r = 0.920, 95% CI for r: 0.685, 0.982) (Table S4).

At the family level, the relative abundance of Lachnospiraceae, Ruminococcaceae, and Rikenellaceae was significantly higher in the *Lb. pentosus*-treated group compared to the control group, with relative abundance of 33.13% vs. 18.87%, 8.59% vs. 4.60%, and 3.31% vs. 1.84%, respectively [(*p* = 0.016, r = 0.920, 95% CI for r: 0.685, 0.982), (*p* = 0.032, r = 0.840, 95% CI for r: 0.439, 0.962), (*p* = 0.008, r = 1, 95% CI for r: 1, 1), respectively] (Figure 3B, Table S4). Conversely, families such as S24_7 and Lactobacillaceae were found at lower relative abundance in the LB group (21.67% vs. 36.00% and 2.19% vs. 5.95 %, respectively) (*p =* 0.056, r = −0.760, 95% CI for r: −0.941, −0.241), while Desulfovibrionaceae showed an increase (1.27% vs. 0.68%) (*p =* 0.056, r = 0.760, 95% CI for r: 0.241, 0.941). These changes, while indicative of a shift in the microbial community, were only marginally significant (Figure 3B, Table S4).

### 3.4. Analysis of GI Microbial Communities

At 97% similarity threshold, an analysis of the V4 amplicon of the *16S rRNA* from colonic, cecal, and ileal samples identified 63, 58, and 72 OTUs at the family level, respectively. Following the application of a 0.1% abundance filter and a 25% prevalence rule, 28, 29, and 24 OTUs were retained for the colon, cecum, and ileum, respectively (Tables S5–S7), with 35, 29, and 48 low-abundance OTUs excluded. Rarefaction analysis demonstrated that sequencing depth was sufficient (Figures S6–S8).

**Figure 4:**
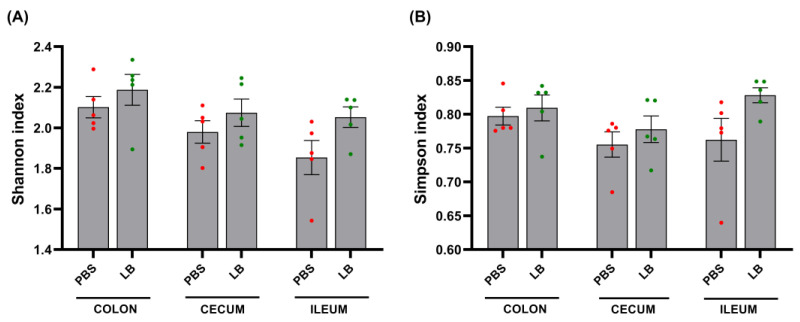
Shannon (**A**) and Simpson (**B**) diversity indices at end of intervention. Data are presented as mean ± SEM. PBS: control group; LB: *Lb. pentosus* PE11 group. *p* > 0.05, not indicated.

**Figure 5:**
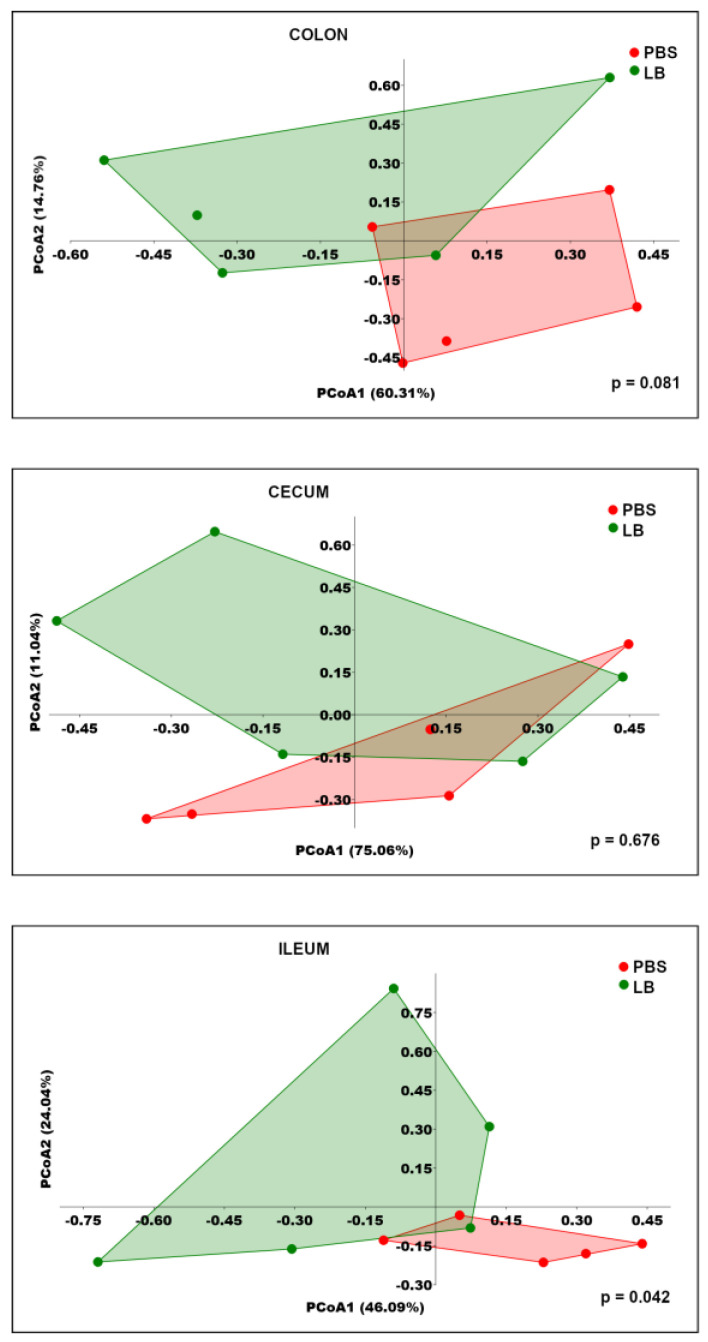
PCoA based on Bray–Curtis distance for colonic, cecal, and ileal microbiota after six-week intervention. Numbers in brackets indicate percentage of variance explained by corresponding coordinates (PCoA1 and PCoA2). *p*-value was calculated using PERMANOVA. Each point on graph represents microbial community of sample of individual mouse.

Alpha diversity, assessed using Shannon and Simpson indices, did not significantly differ between the groups across the three sites (Figure 4). PCoA based on Bray–Curtis distances showed no significant differences in the structures of the colonic and cecal microbial communities between the PBS and LB groups (Figure 5). However, the ileal bacterial community exhibited a significant change, suggesting an alteration in the relative abundance of specific bacterial taxa during the intervention (Figure 5).

**Figure 6:**
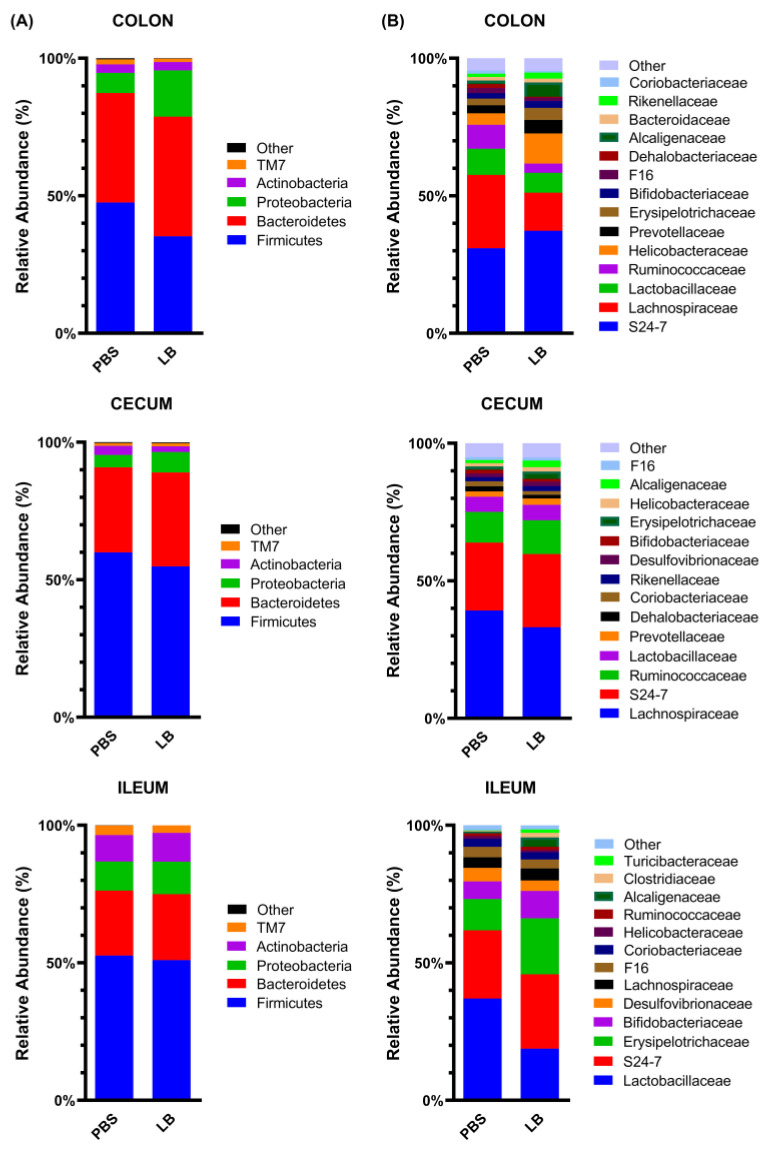
Relative abundance graphs of bacterial OTUs at phylum (**A**) and family (**B**) levels along GI tract at end of intervention. “Other” represents bacterial taxa with median relative abundance of less than 1% in both groups. PBS: control group; LB: *Lb. pentosus* PE11 group.

In the GI track microbial communities, the phyla Firmicutes and Bacteroidetes were the most dominant across all regions, comprising over 73% of the total bacterial population in the colon, 83% in the cecum, and 63% in the ileum. Proteobacteria accounted for 5.98–23.17% in the colon, 3.86–8.93% in the cecum, and 2.36–23.77% in the ileum, while Actinobacteria contributed 1.17–7.33% in the colon, 1.28–5.97% in the cecum, and 6.85–20.16% in the ileum. Statistical analysis revealed no significant differences in the median relative abundances of these four dominant phyla between the PBS and LB groups across all regions (Figure 6A; Tables S5–S7).

In the colon of the LB group, an increased abundance of the Rikenellaceae family was detected, while F16 and Dehalobacteriaceae families exhibited lower abundances with relative abundances of 2.02% vs. 1.10%, 1.14% vs. 1.73%, and 0.34% vs. 1.32%, respectively [(*p* = 0.032, r = 0.840, 95% CI for r: 0.439, 0.962), (*p* = 0.024, r = −0.840, 95% CI for r: −0.962, −0.439), (*p* = 0.008, r = −1, 95% CI for r: −1, −1), respectively] (Figure 6B, Table S5). Moreover, no significant differences in the relative abundances of cecal families were observed between the LB group and the PBS group (Figure 6B, Table S6). In the ileum of the animals of the LB group, however, Lactobacillaceae, the most predominant family in the ileal microbial community of mice, was detected at a significantly lower abundance compared to the PBS group (15.18% vs. 32.68%) (*p* = 0.016, r = −0.920, 95% CI for r: −0.982, −0.685) (Figure 6B, Table S7). Furthermore, the median relative abundance of Erysipelotrichaceae and Alcaligenaceae was higher in the LB group compared to the PBS group (16.45% vs. 10.03% and 2.78% vs. 0.54%, respectively) (*p* = 0.056, r = 0.760, 95% CI for r: 0.241, 0.941). These differences, however, were marginally significant (Figure 6B, Table S7).

### 3.5. Gene Expression Analysis

**Figure 7:**
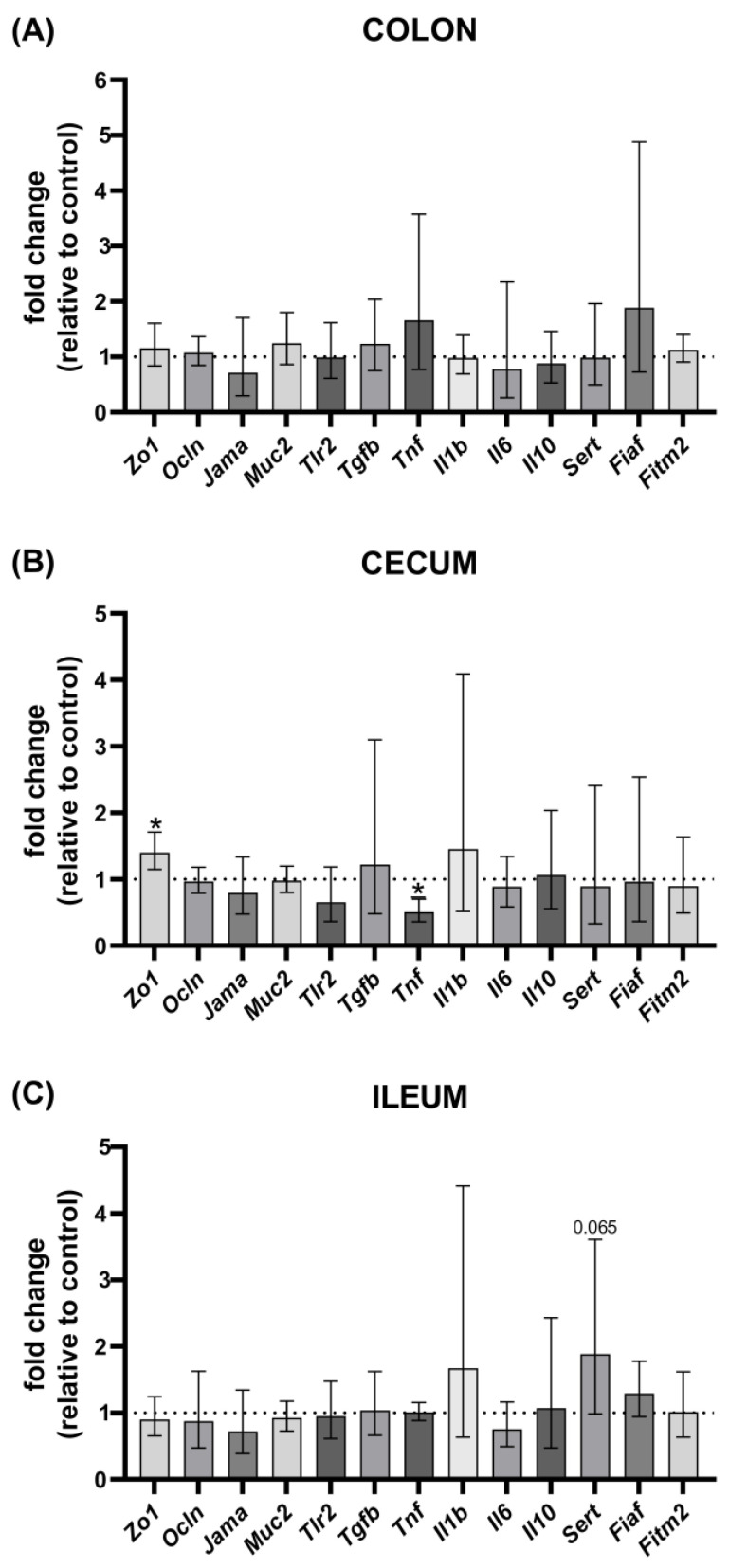
Effect of *Lb. pentosus* PE11 on relative gene expression in colon, cecum, and ileum following six-week dietary intervention. Fold change for each gene is represented by individual bars. Data are expressed as geometric mean with 95% CI. * *p* < 0.05, *p* > 0.05 value not indicated.

We also analyzed, in distinct parts of the GI track (colon, cecum, and ileum), the expression of markers related to the integrity of the gut barrier (*Zo1, Ocln*, *Jama*), mucin expression (*Muc2*), serotonin (*Sert*), and lipid (*Fiaf*, *Fitm2*) metabolism. Furthermore, we evaluated the expression of genes linked to immune system regulation, as genes coding the toll-like receptor 2 (*Tlr2*) and several cytokines (*Tgfb*, *Tnf*, *Il1b*, *Il6*, *Il10*) As shown in Figure 7B, the expression of *Zo1* mRNA in the cecum was significantly upregulated in the LB group, with a fold change of 1.40 (*p* = 0.039, d = −1.562, 95% CI for d: −2.977, −0.077), indicating enhanced epithelial barrier integrity. Additionally, *Lb. pentosus* PE11 administration led to a notable reduction in *Tnf* mRNA expression levels in the cecum, with a fold change of 0.50 (*p* = 0.038, d = 1.574, 95% CI for d: 0.086, 2.992) (Figure 7B), suggesting anti-inflammatory effects. The expression of *Sert* mRNA in the ileum exhibited an upward trend in the *Lb. pentosus* PE11-treated group compared to the control group, with a fold change of 1.89. This increase approached statistical significance (*p* = 0.065, d = −1.349, 95% CI for d: −2.717, 0.083) (Figure 7C), indicating a potential modulation of serotonin reuptake in the ileum by *Lb. pentosus* PE11.

### 3.6. Serum Biochemical Parameters

The impact of *Lb. pentosus* PE11 administration on common biochemical parameters used to assess overall health is summarized in Table 1 (values for each animal are presented in Table S8). *Lb. pentosus* PE11 administration had no significant effect on BUN, CRE, HDL, LDL, or ALP. TG and ALT levels, however, were significantly reduced in the mice of the LB group compared to the PBS group (57 mg/dL vs. 91 mg/dL and 33 U/L vs. 75 U/L, respectively) (*p* = 0.008, r = −1, 95% CI for r: −1, −1). Notably, TC levels showed a trend toward reduction in the *Lb. pentosus* PE11 group (67 mg/dL vs. 81 mg/dL) (*p* = 0.056, r = −0.760, 95% CI for r: −0.941, −0.241).

## 4. Discussion

Several studies have demonstrated the beneficial effects of probiotics in managing various diseases, particularly in conditions such as GI disorders, inflammatory diseases, and metabolic disorders. However, their impact on healthy individuals is less well studied, and despite the variety of probiotics available in the market, clear guidelines for their use in healthy populations are still lacking [13]. As a result, interest in this area is increasing. In this study, we investigated the role of a novel potential probiotic strain, *Lb. pentosus* PE11, in healthy mice over a six-week period, focusing on its effects on GI microbiota and markers of overall health.

We first analyzed the impact of *Lb. pentosus* PE11 on the structure of the fecal microbiota. β-diversity and PERMANOVA analyses revealed a clear difference between the structures of the two communities, with shifts in the relative abundance of specific bacterial taxa. More specifically, the F/B ratio increased, with the Lachnospiraceae, Ruminococcaceae, and Rikenellaceae families becoming more abundant in the LB group.

The F/B ratio is commonly used in assessing fecal microbiota structure and was linked to various health conditions. For instance, a decreased F/B ratio was associated with obesity, DSS-induced colitis, or IBD in both mouse models and human patients [24]. The phylum Firmicutes is often associated with anti-inflammatory properties and metabolic regulation, such as improved energy harvesting, enhanced lipid metabolism, and increased insulin sensitivity [25]. Nevertheless, numerous studies have shown that Bacteroidetes bacteria possess pro-inflammatory properties, impacting cytokine production and therefore playing a role in the development of IBD [24]. However, as the F/B ratio varies across populations, age groups and gender in humans or strain in experimental animals, and is influenced by environmental and genetic factors, conflicting findings were reported [24]. Additionally, other phyla, such as Proteobacteria, play significant roles, while only a limited number of probably distinct bacterial species within the Firmicutes and Bacteroidetes phyla, are linked to different conditions or a healthy microbiome.

Several studies have shown that Lachnospiraceae and Ruminococcaceae are key producers of short-chain fatty acids (SCFAs), serving as the primary producers of butyrate [26,27,28,29], one of the most crucial metabolites for host health [30]. Butyrate is implicated in the preservation of gut integrity during inflammation by promoting the expression of tight junction proteins as well as in the protection against *Clostridium* *difficile* colonization and/or *C. difficile* infection [28,30,31]. Interestingly, in alcohol-induced liver disease models, a reduced abundance of the butyrate-producing families, Lachnospiraceae and Ruminococcaceae, was linked not only to decreased butyrate levels but also to the progression of the disease [29]. Moreover, the Lachnospiraceae family was associated with improved lipid metabolism in mouse models of a high-fat diet [32,33]. Members of the family Rikenellaceae are known hydrogen producers with the capability to neutralize cytotoxic reactive oxygen species (ROS). Research indicates that hydrogen can mitigate oxidative stress and suppress key pro-inflammatory cytokines in inflamed tissues, thereby alleviating symptoms of diseases such as IBD [34]. The observed increase in the relative abundance of Rikenellaceae in our study suggests that *Lb. pentosus* PE11 may contribute to reducing oxidative stress, even in healthy animals. An increased abundance of Lachnospiraceae, Ruminococcaceae, Rikenellaceae, and Lactobacillaceae was positively correlated with greater ileal and colonic villus height, indicating a potential link between microbial populations and gut morphology [35]. However, in our study, no statistically significant differences were observed in the height and depth of the gastrointestinal villi and crypts, respectively, despite changes in microbial composition. This suggests that the relationship between gut microbiota and intestinal mucosa morphology may be influenced by additional factors or require a longer intervention period to manifest measurable effects.

The GI tract is a multi-organ system segmented into distinct regions that break down and metabolize food, absorb nutrients, vitamins and minerals, and excrete waste materials. Each region exhibits unique gene expression and functionality, coordinating diverse digestive, immune, metabolic and hormonal activities, which results in region-specific physicochemical conditions within the gut [1]. Since environmental conditions influence the survival and proliferation of microorganisms along the GI tract, each area hosts a distinct gut microbiota composition [2,36,37]. Therefore, the observed effects of probiotics on the gut microbiome and host gene expression are not only strain- and dose-dependent but also site-dependent [38,39]. In this vein, we analyzed the luminal microbial communities and the gene expression across three distinct anatomical and physicochemical regions of the gastrointestinal tract.

The ileal microbial community was influenced by *Lb. pentosus* PE11; Lactobacillaceae abundance was decreased. Lactobacillaceae abundance was also reduced in feces, although this change was not statistically significant, suggesting that the observed change in the fecal microbiota may have arisen from changes in the ileal microbial community. Similar results were obtained in studies in mice fed a high-fat diet [16,40,41], as well as in healthy mice following a five-day intervention with a potential probiotic strain [38]. In addition, in the ileum, gene expression analysis indicated a trend toward increased *Sert* expression. Serotonin has a key role in the regulation of fluid secretion, GI motility, and sensory perception and its elevated expression is often associated with disturbances in gut sensation and motility, which are symptoms observed in various GI disorders, both organic and functional [42]. Sert is responsible for the reabsorption of serotonin by enterocytes in the mucosa, thus maintaining stable serotonin levels within the intestine [43]. Increased *Sert* expression leads to reduced serotonin levels [44,45,46], which may help regulate gut motility and alleviate GI disorder symptoms.

The structure of the microbial communities in the colon was influenced by *Lb. pentosus* PE11; the relative abundance of Rikenellaceae was higher, while the abundances of F16 and Dehalobacteriaceae were reduced. In the fecal samples, the Rikenellaceae family showed a similar increase, alongside higher abundances of Lachnospiraceae and Ruminococcaceae. This suggests that while *Lb. pentosus* PE11 broadly impacts microbial populations in the colon, only certain families demonstrate consistent changes in both colonic and fecal communities.

Despite the fact that *Lb. pentosus* PE11 did not affect the microbial community of the cecum, gene expression analysis revealed decreased *Tnf* expression, as well increased *Zo1* expression in this part of the GI track. TNF, a multifunctional pro-inflammatory cytokine, through the activation of the NF-kB pathway, causes disruption of the intestinal barrier integrity, increasing permeability and enabling luminal antigen penetration. This process amplifies inflammation and plays a central role in intestinal disorders, including IBD [47]. A low-grade inflammatory condition by interfering with insulin signaling and inducing insulin resistance also plays a crucial role in obesity and diabetes [48]. Zo1 is a protein involved in the development of tight junctions and in the maintenance of the intestinal barrier preventing harmful substances from entering in the submucosa and preserving immune homeostasis in the gut [49].

Region-specific biological effects of probiotics on healthy animals were previously documented. For instance, Taverniti et al. [38] showed that probiotics modulate gut microbiota composition and influence immune and serotonergic gene expression in a site-specific manner, with the ileum exhibiting the most pronounced changes. Similarly, a study on *Clostridium butyricum* in weaning Rex rabbits revealed that probiotic supplementation improved growth, immune function, and microbiota composition, with the effects varying by both dose and intestinal region [39]. In our study, the observed changes in gene expression in animals treated with the *Lb. pentosus* PE11 strain suggest localized effects, an enhancement of tight junctions, and a reduction in inflammation, specifically in the cecum. Unlike the human GI where the colon serves as the primary site of fermentation, this process predominantly occurs in the fully developed cecum of rodents, underscoring the importance of this region in mediating probiotic effects.

When markers of overall health were analyzed following the administration of *Lb. pentosus* PE11, a significant decrease in TG and ALT levels was observed, along with a marginal decrease in TC levels. Elevated TG and TC levels are associated with disrupted lipid metabolism, impaired energy balance, and the development of insulin resistance, contributing to fat accumulation and inflammation in the liver and playing a role in the promotion of the atherosclerotic plaque formation [50,51]. The beneficial effects of probiotics in lowering TG levels were mainly demonstrated in interventions involving high-fat diets [16,52,53,54,55,56,57]; however, there are few studies on healthy animals [58,59,60,61]. Regarding the marginally significant decrease in TC, it is calculated by summing LDL cholesterol, HDL cholesterol, and one-fifth of the TG levels. As no differences in LDL and HDL levels were observed, the marginally significant decrease in TC likely reflects the reduction we observed in the TG levels.

ALT, an enzyme primarily found in the liver, is typically present in the bloodstream at low levels and serves as a key marker of liver function. Elevated serum ALT levels indicate liver cell damage, which may result from increased hepatic cell membrane fragility or cell death. Liver damage can have profound effects on metabolic health due to the organ’s central role in metabolism, like impaired glucose regulation and lipid metabolism, contributing to elevated TG and TC levels and insulin resistance [62]. Other studies have reported similar findings following the administration of probiotics, both in healthy individuals [60,61] and in patients with liver diseases [57,63,64,65,66].

Interestingly, in animals treated with *Lb. pentosus* PE11, TG and ALT levels, although decreased, remained within physiological ranges, suggesting that such changes reflect a modulation toward optimal metabolic homeostasis rather than a pathological shift. Serum TG and ALT levels, even within normal ranges, are still associated with an increased risk of cardiovascular, metabolic, and liver diseases over time; levels that fall within the higher end of the normal range, especially in the high normal spectrum, are linked to a greater risk of these conditions [50,62,67,68]. Therefore, reductions in these parameters in healthy individuals, along with a shift toward the lower end of the normal range, may indicate a preventive benefit, promoting overall metabolic and liver health. The ability of *Lb. pentosus* PE11 to lower these parameters, even in healthy individuals, may contribute to the prevention of cardiovascular, metabolic, and liver diseases. This suggests that the benefits of probiotics go beyond symptom management, offering potential for proactive disease prevention in healthy populations. While further research is needed to validate and fully understand the long-term implications of these findings in disease prevention, the current results align with growing evidence that probiotics can positively influence key biomarkers, even in the absence of overt disease.

## 5. Conclusions

The administration of *Lactiplantibacillus pentosus* PE11 in healthy mice over six weeks revealed promising potential as a probiotic, particularly in modulating gut microbiota and improving health markers. Changes in the fecal microbial community structure included an increase in butyrate-producing families like Lachnospiraceae and Ruminococcaceae. Region-specific effects along the gastrointestinal tract were also observed; in the ileum, changes in the microbial community structure and a trend toward increased *Sert* expression indicated a potential role in gut motility regulation, while in the cecum, decreased *Tnf* expression and increased *Zo1* expression point to enhanced intestinal barrier integrity and anti-inflammatory effects. At the systemic level, modest reductions in TG and ALT levels, both within normal ranges, pointed to potential benefits for lipid metabolism and liver health. These findings align with growing evidence linking probiotics to gut homeostasis and immune modulation. In contrast to most studies, which focus on diseased populations, our research observed these effects in healthy individuals, highlighting the potential of probiotics for maintaining overall health and paving the way for clinical studies to establish their utility and guidelines for use in healthy populations.

## Figures and Tables

**Table 1:** Effect of *Lb. pentosus* PE11 administration on biochemical parameters following dietary intervention. Data are expressed as median (min–max).

Biochemical Parameter	PBS Group Control	LB Group *Lb. pentosus* PE11	*p*-Value
ALT (U/L)	75 (53–145)	33 (17–43)	**0.008**
ALP (U/L)	76 (60–96)	87 (78–93)	0.286
BUN (mg/dL)	43 (39–92)	42 (37–47)	0.413
CRE (mg/dL)	0.31 (0.27–0.39)	0.29 (0.28–0.31)	0.460
HDL (mg/dL)	54 (33–66)	40 (39–57)	0.278
LDL (mg/dL)	5 (4–8)	5 (4–6)	0.762
TC (mg/dL)	81 (72–100)	67 (65–94)	0.056
TG (mg/dL)	91 (79–101)	57 (37–67)	**0.008**

## Data Availability

The data presented in this study are available in Tables S2–S8.

## Supplementary Materials

The following supporting information can be downloaded at: https://www.mdpi.com/article/10.3390/nu17030442/s1. Figure S1: Effect of *Lb. pentosus* PE11 on body weight over intervention; Figure S2: Histological evaluation of colonic, cecal, and ileal morphology; Figure S3: Assessment of crypt depth and villus height in control and *Lb. pentosus* PE11-treated mice; Figure S4: Rarefaction and Shannon curves of fecal samples collected at start of dietary intervention; Figure S5: Rarefaction and Shannon curves of fecal samples collected at end of dietary intervention; Figure S6: Rarefaction and Shannon curves of colonic samples collected at end of dietary intervention; Figure S7: Rarefaction and Shannon curves of cecal samples collected at end of dietary intervention; Figure S8: Rarefaction and Shannon curves of ileal samples collected at end of dietary intervention; Table S1: Primers used in qPCR analysis; Table S2: Body weight measurements during dietary intervention; Table S3: OTU tables (feces) at Day 0; Table S4: OTU tables (feces) at end of intervention; Table S5: OTU tables (colon) at end of intervention; Table S6: OTU tables (cecum) at end of intervention; Table S7: OTU tables (ileum) at end of intervention; Table S8: List of values of biochemical parameters of each animal at end of intervention.
