# Supplementary material for: Enhancing Gut Microbiome and Metabolic Health in Mice Through Administration of Presumptive Probiotic Strain Lactiplantibacillus pentosus PE11
Source: Nutrients. 2025 Jan 25;17(3):442. doi: 10.3390/nu17030442 (PMC11820638; doi:10.3390/nu17030442)
Supplement: Supplementary file 1 [file nutrients-17-00442-s001.zip › nutrients-3425378-supplementary.pdf]

Supplementary Materials

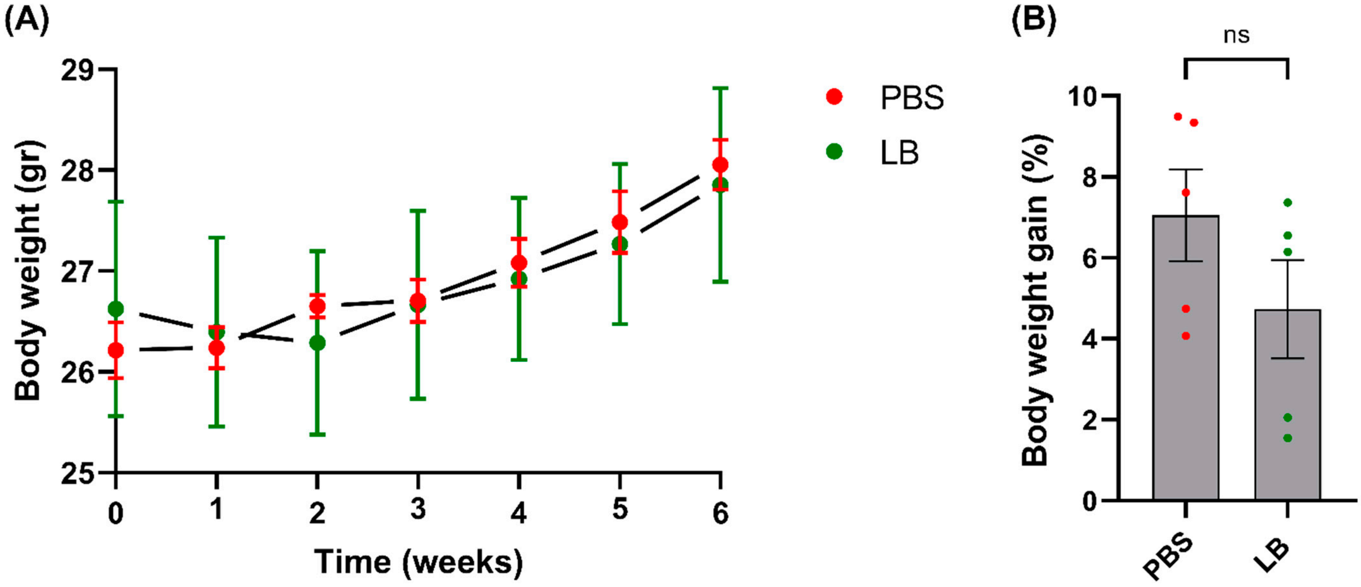

Figure S1. Effect of *Lb. pentosus* PE11 on body weight over the six-week intervention (A) and on the total body weight gain (%) (B). No significant differences were observed. Data are expressed as mean ± SEM. PBS: control group, LB: *Lb. pentosus* PE11-treated group. ns: not significant.

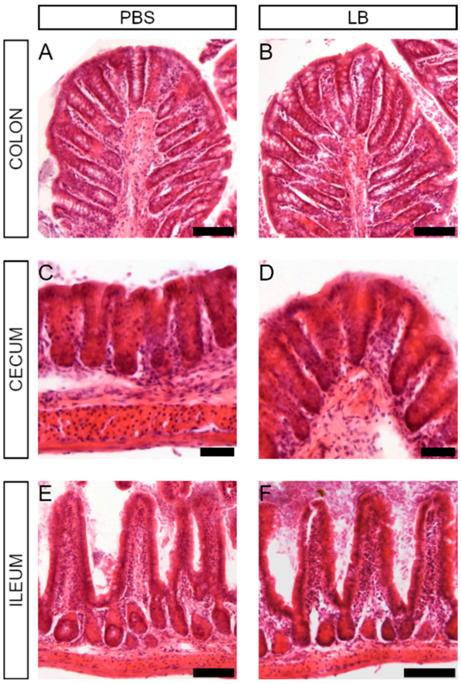

Figure S2. Histological evaluation of colonic (A,B), cecal (B,C), and ileal (D,E) morphology at the end of the intervention. Scale bars in (A,B,E,F): 100 μm, in (C,D): 50 μm. PBS: control group, LB: Lb. pentosus PE11-treated group.

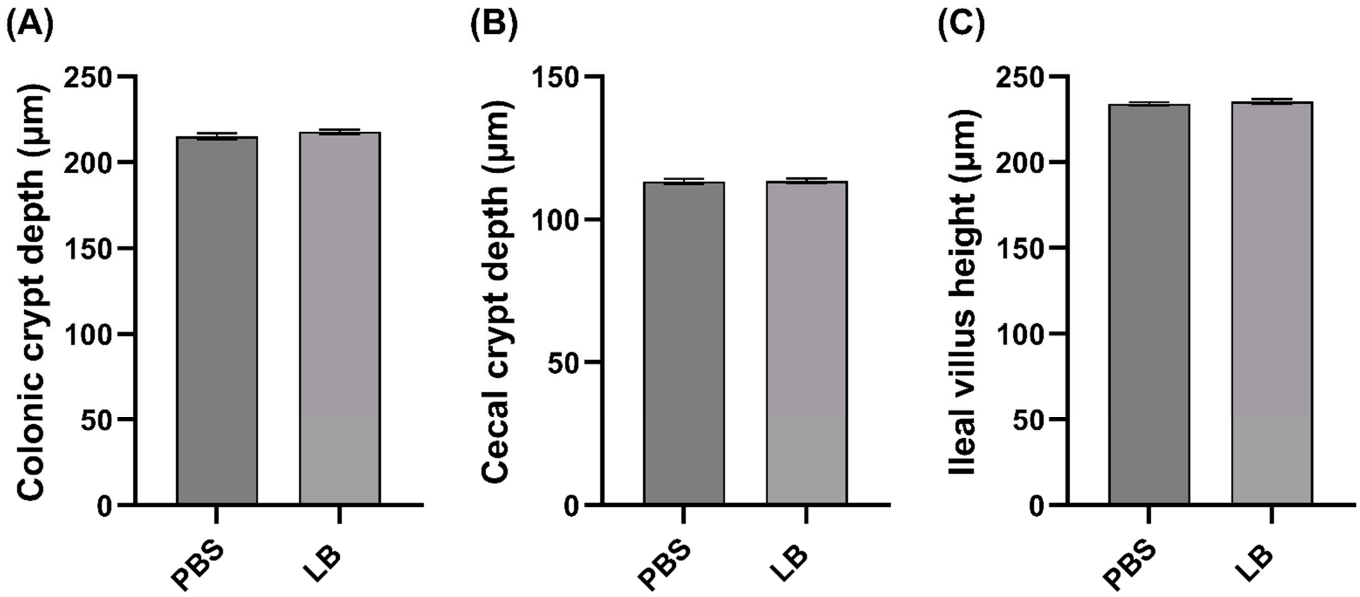

Figure S3. Effect of Lb. pentosus PE11 on colonic (A) and cecal (B) crypt depth and on ileal villus height (C) over the six-week intervention. No significanct differences were observed. Data are expressed as mean ± SEM. PBS: control group, LB: Lb. pentosus PE11-treated group. p > 0.05 value not indicated.

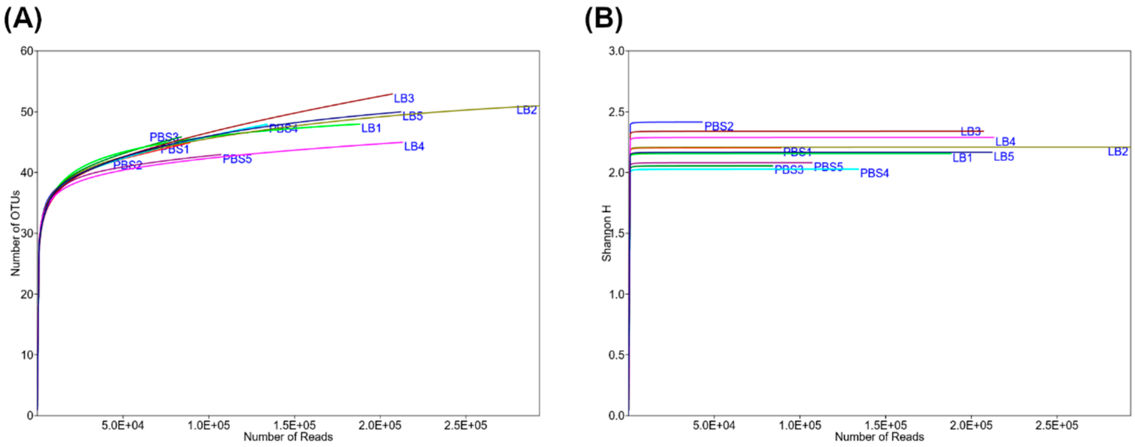

Figure S4. Rarefaction curve (A) and Shannon curve (B) of each fecal sample collected at Day 0. Each line on the graph represents an individual sample. PBS: control group, LB: Lb. pentosus PE11-treated group.

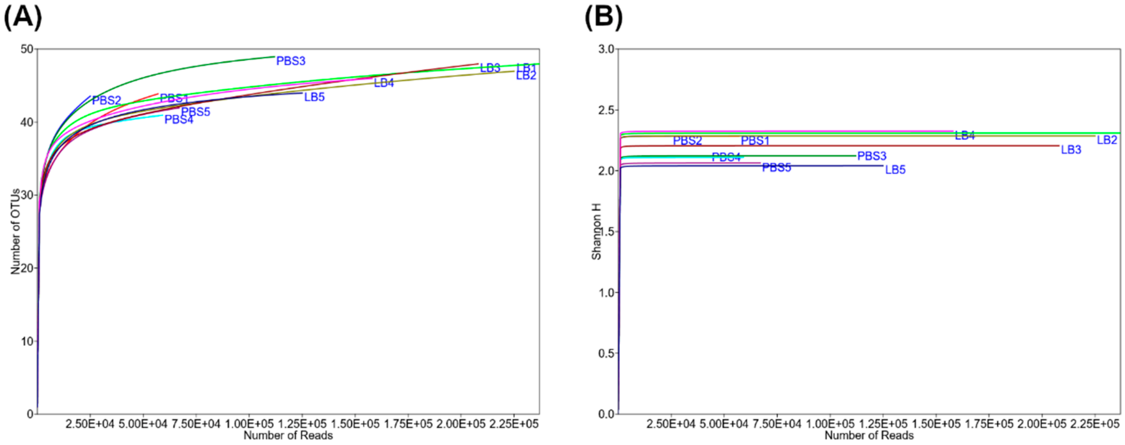

Figure S5. Rarefaction curve (A) and Shannon curve (B) of each fecal sample collected at the end of the intervention. Each line on the graph represents an individual sample. PBS: control group, LB: Lb. pentosus PE11-treated group.

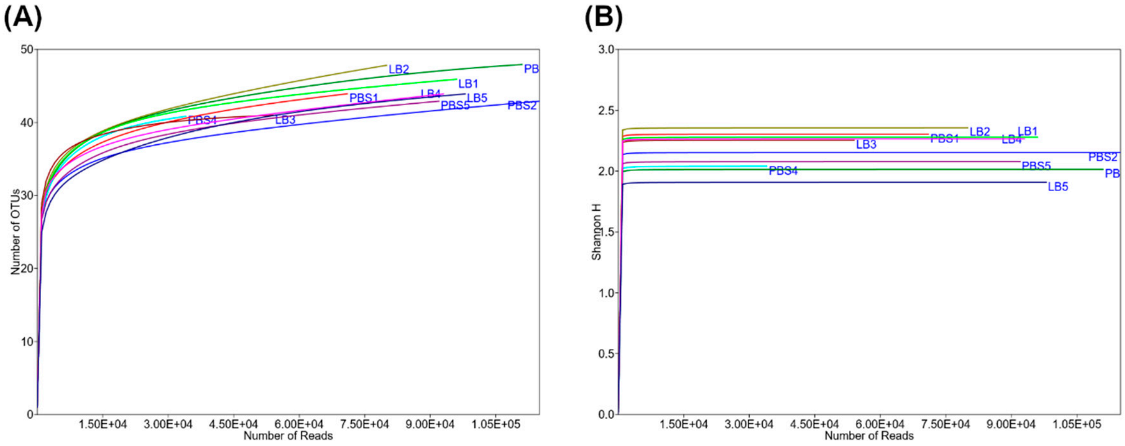

Figure S6. Rarefaction curve (A) and Shannon curve (B) of each colonic sample collected at the end of the intervention. Each line on the graph represents an individual sample. PBS: control group, LB: Lb. pentosus PE 11-treated group.

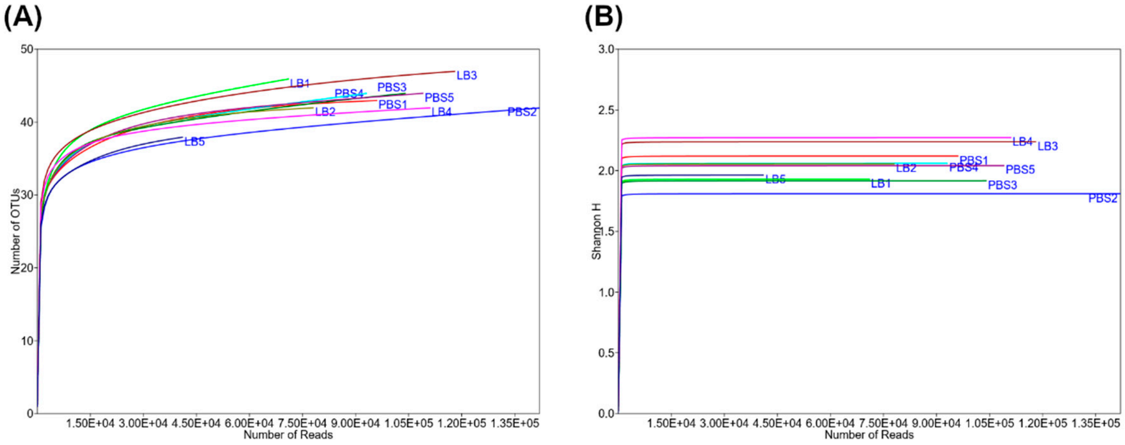

Figure S7. Rarefaction curve (A) and Shannon curve (B) of each cecal sample collected at the end of the intervention. Each line on the graph represents an individual sample. PBS: control group, LB: Lb. pentosus PE11-treated group.

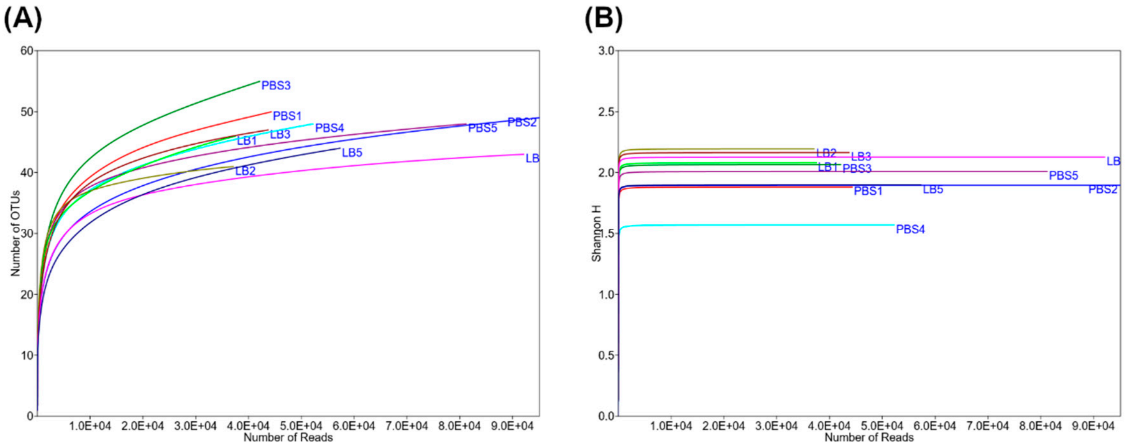

Figure S8. Rarefaction curve (A) and Shannon curve (B) of each ileal sample collected at the end of the intervention. Each line on the graph represents an individual sample. PBS: control group, LB: Lb. pentosus PE11-treated group.

Table S1. List of primer sequences used in qPCR to assess gene expression in mouse biopsies collected from the colon, cecum, and ileum.

Primer	Forward (5'-3')	Reverse (5'-3')	Product length (bp)	Tm (ºC)	Accession number
Zo1	AGCTCATAGTTCAACACAGCCTCCAG	TTCTTCCACAGCTGAAGGACTCACAG	142	67	doi:10.1073/pnas.1525474113
Ocln	ACTCCTCCAATGGCAAAGTG	CCCCACCTGTCGTGTAGTCT	249	60	NM_008756.2<br>NM_001360536.1<br>NM_001360537.1<br>NM_001360538.1<br>NM_001360539.1
Jama	TTCCCGAGAACGAGTCCATC	CTCTCCATTGTCCTTCCGGG	200	60	NM_172647.2
Tlr2	TCAAGGAGGTGCGGACTGT	GCATCCTCTGAGATTTGACGCTT	162	67	NM_011905.3
Muc2	TGATTGGCCACACCTGTCAA	GCCATCATCCAGCAGTCCAT	170	63	NM_023566.4
Tgfb	ACGTGGAAATCAACGGGATCA	GTTGGTATCCAGGGCTCTCC	152	67	NM_011577.2
Tnf	CCTTTCACTCACTGGCCCAA	AGGCCATTTGGGAACTTCTCATC	253	63	NM_013693.3<br>NM_001278601.1
Il1b	GCCACCTTTTGACAGTGATGAG	AAGGTCCACGGGAAAGACAC	219	67	NM_008361.4
Il6	CAGTTGCCTTCTTGGGACTGA	GAATTGCCATTGCACAACTCTTTTC	190	63	NM_031168.2<br>NM_001314054.1
Il10	GGTTGCCAAGCCTTATCGGA	AGACACCTTGGTCTTGGAGCTTA	229	60	NM_010548.2
Fiaf	GCCTGTGGTAACGCTTGTCA	GCCAAGAGGTCTATCTGGCTCT	233	63	NM_020581.2
Fitm2	CGCAACGTCCTCAACGTGTATT	GCAGCTGCCTGTATAGTGCT	207	63	NM_173397.4
Sert	GTGTGGTGAACTGCATGACG	CCAGGCCTGCGAACGTACTA	237	63	NM_010484.2
Canx	CCTCTTCTGCTGTTCTGGAAAGAA	GTTCAAAATTTCATCCTCCTCTGCTTTAG	241	63	NM_007597.3<br>NM_001110499.1<br>NM_001110500.1

Table S2. Body weight measurements of each mouse during the intervention. PBS: control group, LB: Lb. pentosus PE11 group.

Body weight (gr)	PBS					LB					*p-value*
**Time (weeks)**	**Animal No1**	**Animal No2**	**Animal No3**	**Animal No4**	**Animal No5**	**Animal No1**	**Animal No2**	Animal No3	Animal No4	Animal No5	
0	27.19	25.72	26.05	25.70	26.41	25.72	24.30	27.46	30.36	25.28	0.7191
1	27.00	26.03	26.02	25.85	26.29	25.88	24.44	27.12	29.66	24.87	0.8749
2	26.97	26.76	26.34	26.47	26.72	25.87	24.90	27.40	29.18	24.09	0.7015
3	27.22	27.21	26.43	26.39	26.27	26.50	24.77	27.63	29.70	24.73	0.9694
4	27.41	27.41	26.18	27.01	27.39	26.67	25.26	27.99	29.42	25.27	0.8552
5	27.89	27.75	26.28	27.58	27.93	26.88	25.84	28.53	29.63	25.46	0.8045
6	28.48	28.16	27.11	28.10	28.42	27.30	26.09	29.26	30.83	25.80	0.8468
Total body weight gain (%)	4.74	9.49	4.07	9.34	7.61	6.14	7.37	6.55	1.55	2.06	0.2009

Table S3. Relative abundance of fecal bacterial OTUs in the two groups of mice at the start of the intervention. PBS: control group, LB: *Lb. pentosus* PE11 group.

Bacterial OTUs	PBS					LB					*p-value*
**Phylum level**	**Animal No1**	**Animal No2**	**Animal No3**	**Animal No4**	**Animal No5**	**Animal No1**	**Animal No2**	Animal No3	Animal No4	Animal No5	
Bacteroidetes	44.03	48.22	23.75	46.88	45.66	54.43	49.88	51.88	34.97	43.37	0.4206
Firmicutes	41.99	40.67	64.14	41.25	40.35	34.45	39.27	31.75	50.56	45.82	0.4206
Proteobacteria	8.61	6.96	5.00	4.71	7.15	6.64	6.81	7.22	9.03	6.74	0.6905
Actinobacteria	4.25	1.62	3.61	4.32	5.12	0.90	1.37	4.78	1.90	1.13	0.1508
TM7	0.76	2.02	3.04	2.72	1.56	2.93	2.38	4.23	2.23	2.72	0.2381
Tenericutes	0.26	0.41	0.21	0.02	0.01	0.48	0.16	0.05	0.05	0.02	>0,999999
Deferribacteres	0.10	0.10	0.24	0.09	0.16	0.16	0.14	0.09	1.27	0.20	0.4921
Firmicutes/Bacteroidetes ratio	0.95	0.84	2.70	0.88	0.88	0.63	0.79	0.61	1.45	1.06	0.3968
Family level											
S24_7	36.38	26.06	17.94	41.66	40.67	36.18	32.86	34.87	26.91	30.61	0.6905
Lachnospiraceae	19.69	15.94	41.07	21.99	17.27	21.58	14.67	14.29	29.22	29.17	0.8413
Erysipelotrichaceae	11.56	3.63	1.17	4.82	5.15	1.26	1.74	5.87	3.46	4.05	0.5476
Ruminococcaceae	4.98	4.42	10.41	5.67	4.31	5.98	3.77	4.31	8.60	9.55	>0,999999
Alcaligenaceae	3.60	0.97	0.67	1.57	1.58	1.36	2.38	2.32	2.17	1.41	0.5476
Lactobacillaceae	3.53	14.30	8.53	6.84	11.28	3.59	17.43	5.61	5.56	0.67	0.4206
Prevotellaceae	3.11	11.61	2.73	2.05	2.05	10.25	10.93	9.31	1.94	4.43	0.5000
Helicobacteraceae	2.97	4.29	3.40	1.52	4.83	4.68	3.78	4.26	4.63	3.26	0.5476
Bifidobacteriaceae	2.71	0.93	2.99	2.72	3.90	0.22	0.60	3.91	0.27	0.58	0.1508
Coriobacteriaceae	1.46	0.64	0.58	1.49	0.47	0.63	0.71	0.80	1.49	0.28	>0,999999
Rikenellaceae	1.26	4.75	1.21	0.89	0.84	3.16	1.72	2.83	2.96	3.17	0.1508
Desulfovibrionaceae	1.24	0.24	0.62	1.44	0.66	0.22	0.25	0.44	1.44	1.76	0.8889
Dehalobacteriaceae	1.22	0.73	1.27	0.88	1.36	0.48	0.33	0.67	1.63	0.87	0.2222
Paraprevotellaceae	0.92	0.09	0.04	0.20	0.08	0.18	0.76	0.33	0.09	0.27	0.3333
Bacteroidaceae	0.89	1.82	0.93	0.80	0.82	1.42	1.34	2.19	1.09	1.50	0.0952
Proteobacteria_unclassified	0.80	1.46	0.32	0.18	0.07	0.38	0.40	0.20	0.79	0.30	>0,999999
F16	0.76	2.02	3.04	2.72	1.56	2.93	2.38	4.23	2.23	2.72	0.2381
Clostridiales_unclassified	0.53	0.52	0.80	0.60	0.51	0.61	0.32	0.37	1.31	0.68	>0,999999
Porphyromonadaceae	0.50	0.46	0.16	0.45	0.49	0.31	0.59	0.39	0.38	0.36	0.5476
Odoribacteraceae	0.50	1.49	0.43	0.47	0.29	1.03	0.61	1.02	1.15	1.20	0.1508
Firmicutes_unclassified	0.26	0.35	0.47	0.17	0.12	0.75	0.36	0.44	0.62	0.43	0.0556
RF39_unclassified	0.26	0.41	0.21	0.02	0.01	0.48	0.16	0.05	0.05	0.02	>0,999999
Bacteroidetes_unclassified	0.24	0.79	0.09	0.13	0.16	0.51	0.43	0.31	0.19	0.54	0.2222
Bacteroidales_unclassified	0.24	1.16	0.22	0.23	0.26	1.39	0.64	0.63	0.25	1.29	0.0952
Mogibacteriaceae	0.11	0.08	0.19	0.12	0.14	0.13	0.10	0.10	0.13	0.15	0.9841
Deferribacteraceae	0.10	0.10	0.24	0.09	0.16	0.16	0.14	0.09	1.27	0.20	0.4921

Turicibacteraceae	0.09	0.06	0.10	0.12	0.11	0.01	0.03	0.03	0.02	0.02	0.0079
Actinobacteria_unclassified	0.08	0.05	0.04	0.11	0.75	0.05	0.06	0.07	0.13	0.27	0.8889
Clostridiaceae	0.01	0.64	0.13	0.03	0.10	0.06	0.52	0.06	0.01	0.22	>0,999999

Table S4. Relative abundance of fecal bacterial OTUs in the two groups of mice at the end of the intervention. PBS: control group, LB: *Lb. pentosus* PE11 group.

Bacterial OTUs	PBS					LB					
**Phylum level**	**Animal No1**	**Animal No2**	**Animal No3**	**Animal No4**	**Animal No5**	**Animal No1**	**Animal No2**	Animal No3	Animal No4	Animal No5	*p-value*
Bacteroidetes	52.41	51.55	52.88	46.54	52.38	22.87	37.45	31.54	34.16	46.15	0.0079
Firmicutes	36.66	34.49	35.30	46.20	39.28	64.96	50.86	55.57	51.15	41.48	0.0159
Proteobacteria	5.59	8.04	7.36	3.84	3.82	4.98	8.01	8.95	8.70	8.29	0.0952
Actinobacteria	4.03	2.87	1.71	1.84	2.20	3.30	1.57	1.83	3.95	1.63	0.5476
TM7	0.97	2.23	2.13	1.27	1.76	3.21	1.41	1.24	1.68	2.18	0.8413
Tenericutes	0.30	0.31	0.24	0.20	0.41	0.17	0.34	0.04	0.07	0.03	0.0952
Deferribacteres	0.05	0.50	0.36	0.12	0.15	0.50	0.37	0.83	0.28	0.25	0.1667
Firmicutes/Bacteroidetes ratio	0.70	0.67	0.67	0.99	0.75	2.84	1.36	1.76	1.50	0.90	0.0159
Family level											
S24_7	36.00	33.72	39.69	33.26	41.15	14.11	24.11	17.63	21.67	38.16	0.0556
Lachnospiraceae	14.44	18.87	13.54	28.33	22.60	38.24	32.53	38.74	33.13	26.25	0.0159
Erysipelotrichaceae	5.17	3.49	2.32	1.26	1.46	2.57	2.29	1.42	3.86	4.28	0.8413
Ruminococcaceae	3.53	4.60	3.40	8.52	6.81	9.73	8.59	11.59	8.11	8.02	0.0317
Alcaligenaceae	1.88	1.78	2.84	1.12	1.19	0.38	1.40	1.35	2.33	1.70	0.6905
Lactobacillaceae	11.82	5.55	14.19	5.29	5.95	7.42	3.37	1.19	2.19	1.02	0.0556
Prevotellaceae	7.59	11.63	8.81	5.75	4.17	2.41	5.40	4.61	6.20	1.84	0.0952
Helicobacteraceae	2.76	5.35	3.65	1.38	1.23	1.85	1.95	5.52	3.38	4.10	0.5476
Bifidobacteriaceae	3.36	2.27	0.30	0.89	1.43	1.65	0.94	0.69	3.27	0.83	0.8413
Coriobacteriaceae	0.39	0.57	1.34	0.89	0.71	1.51	0.59	1.06	0.64	0.73	0.5476
Rikenellaceae	1.61	1.93	1.46	2.62	1.84	3.12	3.31	3.76	3.40	3.16	0.0079
Desulfovibrionaceae	0.30	0.42	0.68	0.74	0.72	1.27	0.85	1.42	1.71	0.57	0.0556
Dehalobacteriaceae	0.67	0.61	0.54	1.59	0.93	0.95	1.11	1.28	1.59	0.75	0.1587
Paraprevotellaceae	1.50	0.69	0.06	0.13	0.26	0.11	0.13	0.17	0.03	0.15	0.2381
Bacteroidaceae	2.22	1.07	1.27	2.38	2.65	1.17	1.36	2.19	0.66	0.89	0.1508
Proteobacteria_unclassified	0.65	0.49	0.19	0.60	0.68	1.49	3.81	0.65	1.28	1.91	0.0238
F16	0.97	2.23	2.13	1.27	1.76	3.21	1.41	1.24	1.68	2.18	0.8413
Clostridiales_unclassified	0.50	0.55	0.68	0.45	0.66	1.18	0.72	0.69	0.89	0.58	0.0317
Porphyromonadaceae	2.15	0.46	0.20	0.78	0.60	0.35	0.69	0.54	0.20	0.27	0.3333
Odoribacteraceae	0.39	0.75	0.73	0.64	0.65	0.54	0.88	2.08	1.35	0.98	0.0952
Firmicutes_unclassified	0.30	0.50	0.39	0.48	0.67	0.56	0.35	0.42	0.57	0.36	>0,999999
RF39_unclassified	0.30	0.31	0.24	0.20	0.41	0.17	0.34	0.04	0.07	0.03	0.0952
Bacteroidetes_unclassified	0.40	0.53	0.17	0.58	0.29	0.28	0.45	0.24	0.15	0.41	0.4206
Bacteroidales_unclassified	0.54	0.78	0.49	0.40	0.78	0.78	1.10	0.31	0.49	0.29	0.7381
Mogibacteriaceae	0.13	0.08	0.12	0.14	0.10	0.13	0.11	0.17	0.14	0.13	0.2460
Deferribacteraceae	0.05	0.50	0.36	0.12	0.15	0.50	0.37	0.83	0.28	0.25	0.1667
Turicibacteraceae	0.09	0.11	0.09	0.12	0.09	4.17	1.78	0.02	0.61	0.03	0.6825
Actinobacteria_unclassified	0.27	0.04	0.07	0.06	0.06	0.14	0.03	0.08	0.04	0.07	0.9841
Clostridiaceae	0.01	0.12	0.04	0.02	0.00	0.02	0.02	0.05	0.04	0.05	0.4127

Table S5. Relative abundance of colonic bacterial OTUs in the two groups of mice at the end of the intervention. PBS: control group, LB: Lb. pentosus PE11 group.

Bacterial OTUs	PBS					LB					
**Phylum level**	**Animal No1**	**Animal No2**	**Animal No3**	**Animal No4**	**Animal No5**	**Animal No1**	**Animal No2**	Animal No3	Animal No4	Animal No5	*p-value*
Firmicutes	38.94	53.03	56.37	45.49	41.53	47.78	45.01	22.90	32.78	19.53	0.1508
Bacteroidetes	38.05	31.72	31.67	43.94	45.73	39.33	40.08	54.91	40.49	55.03	0.2222
Proteobacteria	17.28	10.95	7.02	6.00	5.98	10.39	10.31	15.62	18.19	23.17	0.1508
Actinobacteria	4.06	2.18	2.76	2.97	3.69	1.17	2.87	5.36	7.33	1.43	>0,999999
TM7	1.16	1.74	1.73	1.37	2.76	1.14	1.40	1.14	1.10	0.80	0.0238
Deferribacteres	0.28	0.22	0.28	0.10	0.06	0.13	0.16	0.05	0.10	0.04	0.1667
Tenericutes	0.23	0.16	0.17	0.13	0.25	0.07	0.17	0.02	0.02	0.00	0.0397
Firmicutes/Bacteroidetes ratio	1.02	1.67	1.78	1.04	0.91	1.21	1.12	0.42	0.81	0.35	0.2222
Family level											
S24_7	28.29	23.32	26.31	37.73	39.93	21.81	29.87	40.02	34.08	46.27	0.5476
Lachnospiraceae	18.48	34.59	37.27	24.46	20.66	30.86	21.64	8.46	12.62	9.65	0.1508
Helicobacteraceae	14.95	7.53	3.81	3.59	3.36	8.39	6.03	10.01	12.40	17.58	0.1508
Lactobacillaceae	9.94	4.30	6.62	9.02	8.71	2.25	9.21	6.61	6.98	1.11	0.3095
Prevotellaceae	4.83	4.67	2.71	2.50	2.00	7.88	4.51	7.74	3.48	1.85	0.5476
Ruminococcaceae	4.63	9.00	8.88	7.81	7.96	8.87	5.19	3.05	2.66	2.23	0.0952
Erysipelotrichaceae	3.90	2.29	0.99	2.04	2.21	0.77	4.01	3.49	8.86	5.67	0.2222
Bifidobacteriaceae	3.05	1.37	0.18	1.79	2.30	0.66	2.23	3.96	5.03	0.82	0.6905
Alcaligenaceae	1.29	1.28	1.16	1.13	1.11	0.74	2.42	5.04	4.76	5.19	0.1508
Rikenellaceae	1.20	1.98	1.04	1.10	1.03	3.82	2.02	1.88	1.41	3.25	0.0317
F16	1.16	1.74	1.73	1.37	2.76	1.14	1.40	1.14	1.10	0.80	0.0238
Bacteroidaceae	1.16	0.33	0.69	1.32	1.44	2.16	1.09	3.03	0.60	1.24	0.5476
Porphyromonadaceae	1.11	0.15	0.12	0.31	0.27	0.82	0.65	0.72	0.15	0.32	0.3413
Dehalobacteriaceae	1.05	1.54	1.38	1.32	1.02	0.97	0.77	0.30	0.34	0.29	0.0079
Paraprevotellaceae	0.58	0.07	0.03	0.04	0.07	1.10	0.07	0.21	0.01	0.09	0.5397
Proteobacteria_unclassified	0.57	0.86	0.45	0.39	0.65	0.70	1.46	0.12	0.15	0.23	0.5476
Coriobacteriaceae	0.55	0.71	2.41	1.07	1.22	0.48	0.59	1.33	2.18	0.58	0.6905
Desulfovibrionaceae	0.47	1.27	1.59	0.89	0.87	0.56	0.40	0.45	0.89	0.17	0.0635
Actinobacteria_unclassified	0.46	0.10	0.18	0.11	0.17	0.04	0.04	0.07	0.12	0.04	0.0317
Clostridiales_unclassified	0.43	0.75	0.77	0.36	0.43	0.37	0.36	0.32	0.51	0.15	0.1190
Firmicutes_unclassified	0.36	0.43	0.33	0.37	0.46	0.58	0.52	0.30	0.25	0.30	0.6508
Bacteroidales_unclassified	0.33	0.21	0.24	0.49	0.50	0.51	1.08	0.59	0.21	0.70	0.1032
Odoribacteraceae	0.32	0.70	0.44	0.31	0.34	0.65	0.44	0.45	0.43	0.93	0.1746
Deferribacteraceae	0.28	0.22	0.28	0.10	0.06	0.13	0.16	0.05	0.10	0.04	0.1667
Bacteroidetes_unclassified	0.23	0.29	0.09	0.14	0.15	0.56	0.34	0.25	0.12	0.37	0.1508
RF39_unclassified	0.23	0.16	0.17	0.13	0.25	0.07	0.17	0.02	0.02	0.00	0.0397
Turicibacteraceae	0.13	0.01	0.08	0.08	0.08	3.10	3.27	0.08	0.43	0.01	0.3016
Clostridiaceae	0.03	0.12	0.06	0.03	0.01	0.01	0.04	0.29	0.14	0.11	0.3333

Table S6. Relative abundance of cecal bacterial OTUs in the two groups of mice at the end of the intervention. PBS: control group, LB: Lb. pentosus PE11 group.

Bacterial OTUs	PBS					LB					
**Phylum level**	**Animal No1**	**Animal No2**	**Animal No3**	**Animal No4**	**Animal No5**	**Animal No1**	**Animal No2**	Animal No3	Animal No4	Animal No5	*p-value*
Firmicutes	61.73	72.18	60.62	45.25	46.42	69.62	66.97	53.52	41.63	50.05	>0,999999
Bacteroidetes	26.79	19.16	31.29	45.73	44.02	23.21	22.14	33.28	41.79	39.34	0.8413
Proteobacteria	6.62	5.45	4.53	3.86	4.54	4.43	7.27	5.84	8.93	7.45	0.1508
Actinobacteria	3.61	1.52	2.27	3.75	3.31	1.28	2.00	5.50	5.97	2.02	>0,999999
TM7	0.89	0.78	1.07	1.20	1.38	1.03	1.22	1.56	1.14	1.04	0.5476
Deferribacteres	0.21	0.82	0.15	0.13	0.19	0.40	0.29	0.27	0.52	0.11	0.5476
Tenericutes	0.14	0.09	0.08	0.09	0.14	0.04	0.12	0.02	0.03	0.00	0.0476
Firmicutes/Bacteroidetes ratio	2.30	3.77	1.94	0.99	1.05	3.00	3.03	1.61	1.00	1.27	>0,999999
Family level											
Lachnospiraceae	39.13	52.82	41.76	23.88	25.64	49.40	43.16	29.54	20.25	32.55	>0,999999
S24_7	20.27	14.22	24.66	38.08	37.46	13.95	17.06	26.14	34.64	33.57	0.6905
Ruminococcaceae	11.31	11.25	10.14	9.49	11.13	12.38	12.22	12.09	5.88	7.99	0.6905
Lactobacillaceae	5.53	2.57	4.27	7.60	5.51	2.54	5.50	6.00	6.38	0.96	0.6905
Helicobacteraceae	2.94	2.35	1.18	1.16	0.84	1.87	1.55	1.17	1.88	1.15	>0,999999
Dehalobacteriaceae	2.86	1.26	2.22	1.86	1.57	1.30	1.68	1.80	0.86	0.87	0.1508
Prevotellaceae	2.31	1.91	3.84	1.95	1.79	3.30	1.19	2.28	3.33	1.45	0.8413
Coriobacteriaceae	1.73	0.77	1.92	2.23	1.83	0.64	0.99	2.03	1.95	1.30	0.6905
Bifidobacteriaceae	1.63	0.70	0.18	1.34	1.34	0.58	0.94	3.31	3.92	0.68	0.8016
Erysipelotrichaceae	1.48	1.77	0.58	1.18	1.20	0.69	1.52	2.64	6.25	6.57	0.1508
Proteobacteria_unclassified	1.44	0.76	0.46	0.51	1.35	0.28	3.28	0.33	0.29	1.10	0.4206
Desulfovibrionaceae	1.35	1.52	1.71	1.07	1.20	1.79	1.24	1.97	1.62	0.75	0.5476
Rikenellaceae	1.26	1.72	1.06	2.04	1.47	2.46	1.78	1.71	1.98	1.80	0.1508
Porphyromonadaceae	0.98	0.11	0.13	0.52	0.39	0.51	0.30	0.37	0.23	0.22	0.8413
Bacteroidaceae	0.95	0.16	0.78	2.02	1.66	1.36	0.63	1.58	0.69	0.85	0.6905
Clostridiales_unclassified	0.91	1.81	1.03	0.63	0.77	0.93	1.06	0.60	0.94	0.50	0.6905
Alcaligenaceae	0.89	0.82	1.19	1.12	1.15	0.49	1.20	2.37	5.14	4.45	0.1508
F16	0.89	0.78	1.07	1.20	1.38	1.03	1.22	1.56	1.14	1.04	0.5476
Bacteroidetes_unclassified	0.37	0.09	0.12	0.18	0.17	0.26	0.15	0.12	0.12	0.18	>0,999999
Firmicutes_unclassified	0.33	0.48	0.35	0.44	0.38	0.43	0.35	0.37	0.39	0.38	0.9524
Odoribacteraceae	0.33	0.75	0.37	0.49	0.48	0.50	0.66	0.75	0.61	0.67	0.1032
Actinobacteria_unclassified	0.25	0.05	0.16	0.18	0.15	0.05	0.07	0.16	0.10	0.04	0.1508
Deferribacteraceae	0.21	0.82	0.15	0.13	0.19	0.40	0.29	0.27	0.52	0.11	0.5476
Bacteroidales_unclassified	0.20	0.16	0.31	0.24	0.25	0.82	0.35	0.24	0.17	0.52	0.2381
RF39_unclassified	0.14	0.09	0.08	0.09	0.14	0.04	0.12	0.02	0.03	0.00	0.0476
Paraprevotellaceae	0.12	0.03	0.02	0.20	0.35	0.06	0.02	0.09	0.02	0.07	0.3016
Mogibacteriaceae	0.11	0.10	0.12	0.07	0.11	0.08	0.12	0.12	0.12	0.09	0.5556
Turicibacteraceae	0.07	0.01	0.10	0.10	0.09	1.87	1.33	0.09	0.44	0.01	0.2937
Clostridiaceae	0.01	0.10	0.03	0.01	0.01	0.02	0.03	0.26	0.11	0.13	0.0556

Table S7. Relative abundance of ileal bacterial OTUs in the two groups of mice at the end of the intervention. PBS: control group, LB: Lb. pentosus PE11 group.

Bacterial OTUs	PBS					LB					
**Phylum level**	**Animal No1**	**Animal No2**	**Animal No3**	**Animal No4**	**Animal No5**	**Animal No1**	**Animal No2**	Animal No3	Animal No4	Animal No5	*p-value*
Firmicutes	52.51	41.67	47.68	73.13	50.18	61.56	57.99	48.81	39.69	39.21	0.6905
Bacteroidetes	22.58	36.65	19.48	13.74	30.11	21.30	22.96	17.07	24.09	29.12	>0,999999

Proteobacteria	13.94	10.07	13.80	4.34	6.44	2.36	6.73	11.33	21.23	23.77	0.6905
Actinobacteria	9.20	8.10	12.14	7.62	9.63	10.03	7.75	20.16	14.07	6.85	0.6905
TM7	1.74	3.33	6.85	1.08	3.64	4.74	4.56	2.62	0.79	0.91	0.6905
Tenericutes	0.04	0.17	0.05	0.10	0.01	0.01	0.01	0.01	0.13	0.13	0.6032
Firmicutes/Bacteroidetes ratio	2.33	1.14	2.45	5.32	1.67	2.89	2.53	2.86	1.65	1.35	0.8413
Family level											
Lactobacillaceae	38.47	26.23	32.68	57.05	29.31	12.50	28.27	25.04	15.18	5.46	0.0159
S24_7	21.93	35.32	18.70	13.22	29.00	20.21	21.81	16.43	23.82	28.92	0.8413
Desulfovibrionaceae	12.49	4.04	11.91	3.03	4.36	0.57	3.06	9.25	11.38	2.30	0.3095
Erysipelotrichaceae	10.03	13.23	9.13	9.79	11.91	12.48	10.93	16.45	19.83	31.20	0.0556
Bifidobacteriaceae	5.60	5.69	0.48	6.68	6.87	8.60	5.68	14.24	7.99	4.59	0.3095
Coriobacteriaceae	3.37	2.30	10.76	0.85	2.55	1.31	1.89	5.52	5.79	2.13	0.8413
Lachnospiraceae	2.57	1.27	3.38	4.60	7.07	3.56	4.80	3.62	1.69	0.88	0.8413
F16	1.74	3.33	6.85	1.08	3.64	4.74	4.56	2.62	0.79	0.91	0.6905
Helicobacteraceae	0.95	0.89	1.15	0.78	1.35	0.63	0.73	0.58	0.31	9.54	0.1508
Ruminococcaceae	0.59	0.23	1.07	0.88	1.00	1.00	1.22	0.96	0.35	0.18	>0,999999
Streptococcaceae	0.42	0.05	0.33	0.07	0.23	0.12	0.03	0.08	0.02	0.04	0.0952
Alcaligenaceae	0.39	5.07	0.68	0.43	0.54	1.05	2.78	1.45	9.52	11.90	0.0556
Actinobacteria_unclassified	0.23	0.11	0.90	0.10	0.21	0.11	0.17	0.41	0.28	0.13	0.8889
Rikenellaceae	0.20	1.03	0.26	0.19	0.40	0.39	0.49	0.23	0.11	0.05	0.5476
Prevotellaceae	0.16	0.16	0.29	0.20	0.23	0.36	0.31	0.23	0.06	0.05	0.8889
Bacteroidaceae	0.16	0.02	0.12	0.09	0.34	0.23	0.20	0.12	0.03	0.01	0.8889
Bacteroidales_unclassified	0.13	0.10	0.11	0.04	0.14	0.11	0.15	0.06	0.08	0.09	0.7302
Turicibacteraceae	0.12	0.00	0.15	0.14	0.13	31.33	12.08	0.13	0.96	0.01	0.2381
Proteobacteria_unclassified	0.12	0.08	0.05	0.09	0.19	0.12	0.16	0.05	0.02	0.04	0.4206
Firmicutes_unclassified	0.11	0.13	0.15	0.15	0.20	0.31	0.25	0.13	0.10	0.08	0.9127
Dehalobacteriaceae	0.10	0.02	0.11	0.10	0.13	0.12	0.17	0.10	0.02	0.02	>0,999999
Clostridiales_unclassified	0.10	0.06	0.10	0.07	0.18	0.11	0.20	0.09	0.06	0.01	0.8730
Mycoplasmataceae	0.04	0.17	0.05	0.10	0.01	0.01	0.01	0.01	0.13	0.13	0.6032
Clostridiaceae	0.01	0.47	0.56	0.27	0.03	0.02	0.05	2.23	1.46	1.33	0.3095

Table S8. Serum biochemical measurements of each mouse at the end of the intervention. PBS: control group, LB: Lb. pentosus PE11 group.

Biochemical parameters	PBS					LB				
	Animal No1	Animal No2	Animal No3	Animal No4	Animal No5	Animal No1	Animal No2	Animal No3	Animal No4	Animal No5
ALT (U/L)	91	145	75	53	64	28	17	40	43	33
ALP (U/L)	76	72	60	82	96	87	93	78	87	81
BUN (mg/dL)	92	43	39	52	42	37	42	40	47	43
CRE (mg/dL)	0.39	0.27	0.31	0.32	0.29	0.29	0.31	0.3	0.29	0.28
HDL (mg/dL)	51	33	54	66	62	40	39	39	41	57
LDL (mg/dL)	4	8	6	5	5	5	5	4	5	6
TC (mg/dL)	72	81	76	99	100	67	65	65	69	94
TG (mg/dL)	91	86	101	79	91	66	37	55	57	67
